# Identification of proliferative and non-proliferative subpopulations of leukemic cells in CLL

**DOI:** 10.1038/s41375-022-01656-4

**Published:** 2022-07-28

**Authors:** Kirsty M. Cuthill, Yan Zhang, Andrea Pepper, Lies Boelen, Eve Coulter, Becca Asquith, Stephen Devereux, Derek C. Macallan

**Affiliations:** 1 Department of Haematology, King’s College Hospital, London, UK; 2 Institute for Infection and Immunity, St George’s, University of London, London, UK; 3 Brighton and Sussex Medical School, Medical Research Building, University of Sussex, Brighton, UK; 4 Department of Infectious Disease, Imperial College, London, UK; 5 Centre for Cancer Genomics and Computational Biology, Bart’s Cancer Institute, Queen Mary University of London, John Vane Science Centre, Charterhouse Square, London, UK; 6 Department of Haemato-Oncology, Division of Cancer Studies, Faculty of Life Sciences and Medicine, King’s College London, London, UK; 7 Infection Care Group, St George’s University Hospitals NHS Foundation Trust, London, UK

**Keywords:** Chronic lymphocytic leukaemia, Targeted therapies

## Abstract

Pathogenesis in chronic lymphocytic leukemia (CLL) is strongly linked to the potential for leukemic cells to migrate to and proliferate within lymph-nodes. Previous in vivo studies suggest that all leukemic cells participate in cycles of migration and proliferation. In vitro studies, however, have shown heterogeneous migration patterns.

To investigate tumor subpopulation kinetics, we performed in vivo isotope-labeling studies in ten patients with *IgVH*-mutated CLL (M-CLL). Using deuterium-labeled glucose, we investigated proliferation in sub-populations defined by CXCR4/CD5 and surface (sIgM) expression. Mathematical modeling was performed to test the likelihood that leukemic cells exist as distinct sub-populations or as a single population with the same proliferative capacity. Further labeling studies in two patients with M-CLL commencing idelalisib investigated the effect of B-cell receptor (BCR) antagonists on sub-population kinetics.

Modeling revealed that data were more consistent with a model comprising distinct sub-populations (*p* = 0.008) with contrasting, characteristic kinetics. Following idelalisib therapy, similar labeling suppression across all sub-populations suggested that the most proliferative subset is the most sensitive to treatment. As the quiescent sub-population precedes treatment, selection likely explains the persistence of such residual non-proliferating populations during BCR-antagonist therapy. These findings have clinical implications for discontinuation of long-term BCR-antagonist treatment in selected patients.

## Introduction

The clinical course and outcome of chronic lymphocytic leukemia (CLL) varies markedly between patients. Investigation of the biological basis of this heterogeneity has contributed much to our understanding of disease pathogenesis. As for many tumors, genomic abnormalities are key determinants of outcome, however, functional properties of the tumor, especially its capacity to migrate into lymphoid tissues and proliferate in response to B-cell receptor (BCR) derived signals, are also important.

In addition to differences between patients with CLL, there is also evidence for heterogeneity within the leukemic clone within individual patients. Large scale sequencing studies have revealed significant subclonal genomic diversity [1, 2] that evolves over time and in response to therapy [3]. Whether this is accompanied by functional intraclonal diversity, however, remains an open question since phenotype, capacity to migrate, signal and proliferate may all change in response to anatomic location and factors in the tumor microenvironment. For example, leukemic cells within the lymph node (LN) express higher levels of the prognostic marker CD38 than those in the peripheral blood (PB) [4], whilst the chemokine receptor CXCR4 is rapidly downregulated following B-cell receptor signaling, and upregulated in its absence [5].

Intraclonal diversity has been directly studied in vivo using the non-radioactive isotope deuterium, administered as heavy water, to label the DNA of dividing cells in patients with CLL [6, 7]. This technique enables the proliferation rate, lifespan and location of populations and subpopulations of tumor cells to be determined from measurements of isotope enrichment in sorted subsets over time. Labeled cells took several days to appear in the PB, compatible with proliferation within and subsequent release from lymphoid tissues, a finding later confirmed by tissue direct sampling [8]. Analysis of sorted subfractions showed that the CXCR4_lo_/CD5_hi_ subset appeared in the blood first and had the highest deuterium enrichment whilst CD5_int_/CXCR4_int_ and CD5_lo_/CXCR4_hi_ subsets show progressively lower and later labeling. In the light of these observations, it was proposed that CXCR4_lo_/CD5_hi_ cells represent the most recently proliferated migrants from tissue to blood, and that, in the PB, these cells quiesce, re-express CXCR4, downregulate CD5 and then re-enter lymphoid tissue where further proliferation may occur. Since deuterium was eventually found in all sorted subsets, these data suggest that the CLL clone behaves in a homogeneous manner and that all cells are similarly able to participate in cycles of migration and proliferation.

Although this model of CLL biology, in which the leukemic clone is functionally homogeneous in respect of its capacity to migrate and proliferate, fits much of the available data, several observations indicate that function may be heterogeneous within the clone. First, although inhibitors of BCR signalling (BCRi) undoubtedly have a major impact on the size of the leukemic clone, in many patients, small numbers of leukemic cells persist [9] suggesting that not all tumor cells are equally dependent on this pathway for survival. Second, CXCR4_lo_/CD5_hi_ peripheral blood CLL cells expressing a similar phenotype to LN CLL cells show preferential migration across vascular endothelium [10], suggesting the existence of a subpopulation primed for LN entry and proliferation. Finally, long term follow-up of patients who have undergone whole exome sequencing has shown a clear relationship between subclonal complexity and tumor growth characteristics in vivo [11].

In view of the above, we re-examined whether the leukemic clone in CLL is always homogeneous in respect of proliferative potential. The deuterium labeling studies described above used heavy water (^2^H_2_0) which, being administered over several weeks, labels multiple generations of cells. Such studies therefore have a limited capacity to resolve subpopulation evolution over time. To address this issue, we opted to use deuterated-glucose (^2^H_2_-glucose) as a labeling agent. Because it has a very short half-life it labels a cohort of cells dividing over a period of less than a day and allows the progress of that population of cells to be tracked through subsequent phenotypic transitions and migration. Although CLL cells divide slowly, the high precursor enrichments achieved with deuterated-glucose (about one log higher than ^2^H_2_O) allow measurable labeling after a short-pulse label. We have previously used this approach to estimate the lifespan of CLL cells, generating average results very similar to those estimated using ^2^H_2_O [12].

**Fig. 1:**
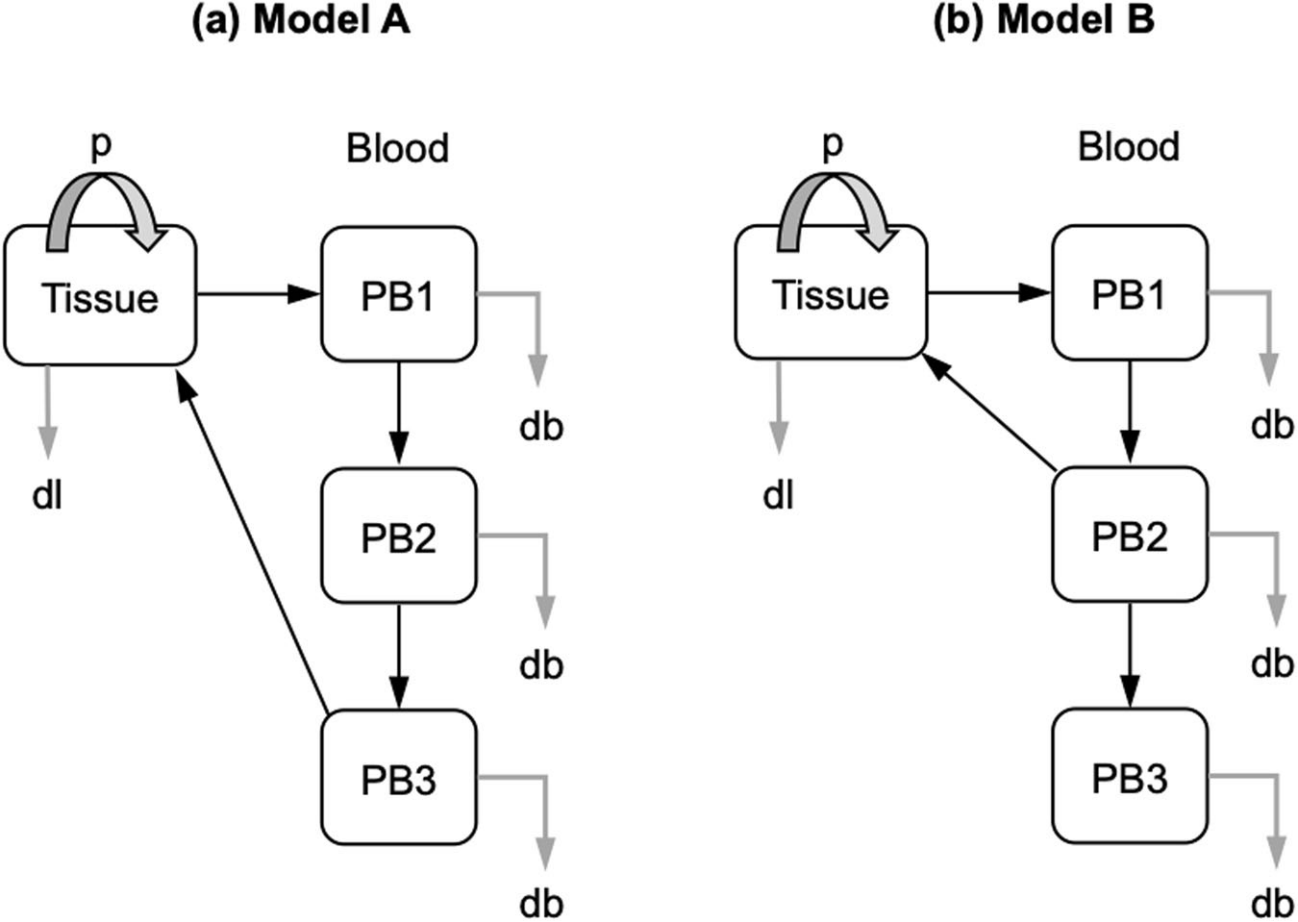
Models for CLL cell proliferation and recirculation. **a** Model A represents the hypothesis that there is a single recirculating pool of cells. We assume all proliferation (p) occurs in a non-blood compartment, denoted “Tissue” and that the blood compartment can be considered as 3 phenotypically distinct subpopulations. Black arrows denote transition between compartments and grey arrows denote cell death or disappearance. **b** Model B represents the alternative hypothesis that there are two (semi-)independent cell populations. The PB2 compartment recirculates to the tissue compartment, leaving PB3 as a distinct non-recirculating pool of cells. Cell disappearance (db, disappearance from blood compartment; dl, disappearance from non-blood compartment is thought (but not assumed) to be primarily due to cell death. Version with all parameters marked is shown in Supplementary Fig. 1.

In the present study, we focused our investigation on patients with M-CLL, the subgroup of patients most likely to exhibit persistent lymphocytosis during BCR inhibitor therapy [9], and therefore most likely to have more readily detectable functional subclonal heterogeneity. Mathematical modeling techniques were then used to determine whether subsets defined by phenotypic markers associated with migration and BCR signalling capacity (CXCR4/CD5, surface IgM [13] and BCR internalization [14]) are more likely to be homogenous, deriving from each other (Model A, Fig. 1), or instead behave as separate subpopulations that remain distinct over time (Model B, Fig. 1). We also report the impact of the BCR antagonist idelalisib on the release and survival of CLL subpopulations labeled prior to and again during therapy.

## Methods

### Patients and sampling

**Fig. 2:**
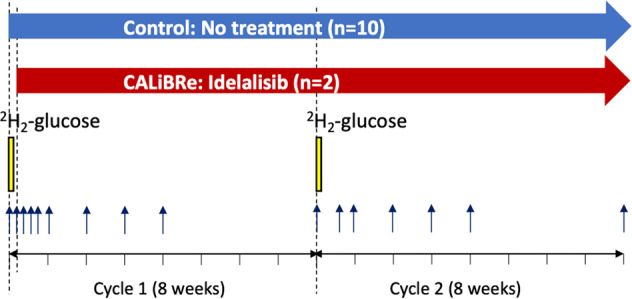
Study schemas. In the observational study, 10 patients underwent labeling with deuterium-labeled glucose (6,6-^2^H_2-_glucose), shown by the yellow bars, on two occasions (Cycles 1 and 2) separated by eight weeks, denoted day 0 and day 56 respectively. In the CALiBRe study, the labeling schedule was performed in an identical way but treatment with idelalisib was commenced on day 1, starting 24 h after commencement of the first labeling cycle, and continued for a minimum of 16 weeks (as indicated by the red arrow). Blood was drawn at regular intervals as shown by the blue arrows (Cycle 1: baseline, daily for the first 4 days, then weekly for 4 weeks; Cycle 2: baseline, day 4, weekly for 4 weeks, then on day 56).

Two cohorts of subjects with CLL with mutated immunoglobulin heavy chain genes (*IGHV*) were studied (Table 1). In the first, 10 treatment-naïve patients with non-progressive Binet Stage A or B M-CLL were labeled with oral deuterium-glucose; PB was drawn and CLL cells isolated at set intervals after labeling (Fig. 2). Duplicate studies were performed 8 weeks later to estimate reproducibility.

**Table 1:** Baseline clinical and laboratory characteristics of patients recruited to deuterium labeling studies.

Trial ID	Age	Gender	Baseline	Binet Stage	*IgVH*	CD38	FISH
	(years)		Lymphocyte		(M/UM)	Expression	
			(x10^9^/L)			(%)	
K1	66	F	7	A	M	7	Normal
K2	55	F	200	B	M	0	13q-
K3	38	F	20	A	M	1	Normal
K4	67	F	60	A	M	0	Normal
K5	62	M	30	A	M	0	Normal
K6	68	M	200	B	M	3	13q-
K7	65	M	90	B	M	0	13q-
K8	69	M	39	B	M	12	Trisomy 12
K9	54	M	100	B	M	0	Normal
K10	53	M	20	A	M	51	Trisomy 12
Cal 001	71	M	222	C	M	7	Normal
Cal 002	85	M	189	C	M	8	13q-

Patients recruited to the CALiBRe study (*n* = 2) are identified by the prefix ‘Cal’.

The second cohort comprised two individuals participating in the CALiBRe study (EudraCT 2012-003631-36) which investigated idelalisib, a phosphoinositide-3 kinase (PI-3K) inhibitor, in patients with previously-untreated symptomatic CLL [15]; deuterium labeling was included as an optional sub-study. CALiBRe closed early due to safety concerns with idelalisib, hence recruitment to the labeling sub-study was limited to two patients. In these subjects, deuterium-glucose administration was timed to begin 24 h before commencing idelalisib treatment (150 mg bd); labeling and sampling matched those in the untreated cohort (Fig. 2). All subjects gave written, informed consent; studies were approved by the UK National Research Ethics Service (13/LO/0434 and 15/YH/0020).

### Deuterium-glucose labeling and analysis

**Fig. 3:**
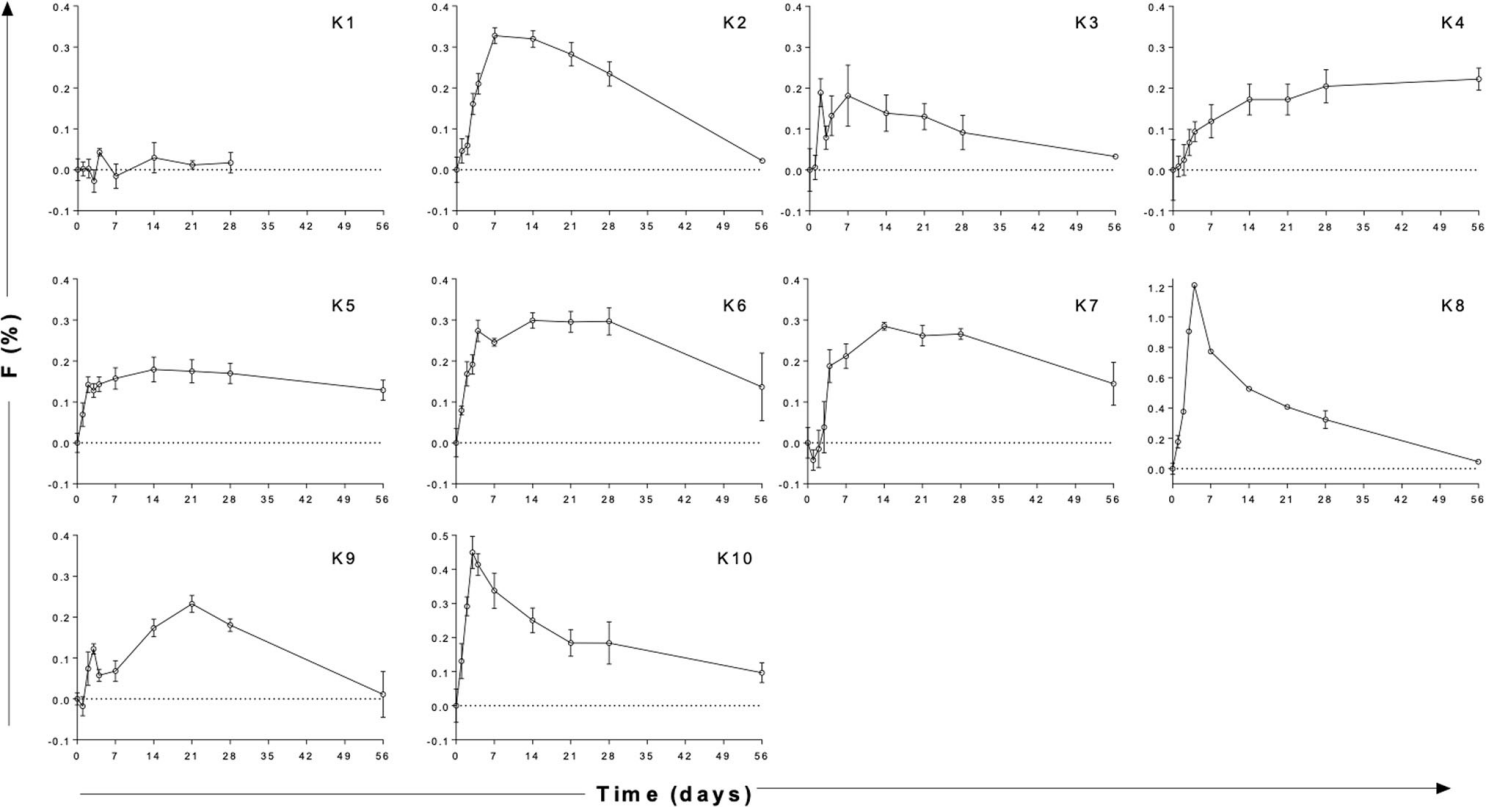
Deuterium labeling in CLL cells in ten patients with non-progressive disease. DNA deuterium enrichments in purified CLL cells from 10 subjects (K1–K10) without intervention. Data are expressed as fraction of new cells produced (F, as a percentage) normalised by the glucose enrichment area under curve to the equivalent of one day’s labeling, i.e. equivalent to %/day. Note different axes for K8 and K10. Cycle 2 results were similar (Supplementary Fig. 2). Time represents days following oral labeling with deuterium-labeled glucose (labeling day = d0); error bars represent the standard deviation of at least four replicate measurements by GC-MS (error bars not shown where they are smaller than the symbols).

**Fig. 4:**
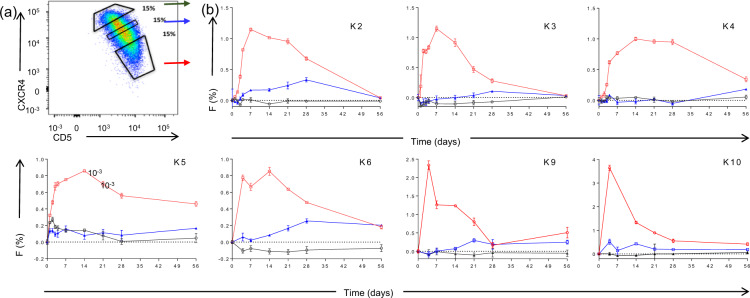
Deuterium labeling for CLL subpopulations defined by CXCR4 and CD5 expression. **a** Sorting protocol for CLL cells according to reciprocal and intermediate expression of CXCR4 and CD5. Gates were set to collect 15% of events within each subpopulation: CXCR4_lo_/ CD5_hi_ (red arrow), CXCR4_int_/CD5_int_ (blue arrow), and CXCR4_hi_/CD5_lo_ (green arrow). **b** Deuterium enrichment curves of sorted CLL cells: CXCR4_lo_CD5_hi_ (red circles), CXCR4_int_CD5_int_ (blue squares), and CXCR4_hi_CD5_lo_ (green triangles). Results are shown from 7 subjects (K2, K3, K4, K5, K6, K9, and K10), expressed as fraction of new cells (F %) normalised to one day’s labeling; error bars represent the standard deviation of ≥4 replicate measurements by GC-MS. The F scale varies between subjects to best represent deuterium enrichment. Time represents days post-labeling. Note that K5 had a dual population of CLL cells.

**Fig. 5:**
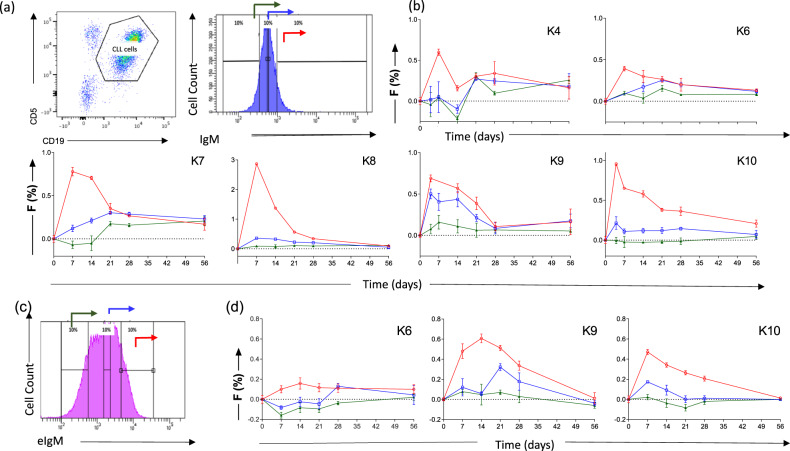
Association of CLL kinetics with surface IgM (sIgM) expression and expression within acidified endosomes (eIgM). **a** Sorting protocol for CLL cells according to sIgM expression. Gates were set to include 10% of events within each subpopulation: sIgM hi (red arrow), sIgM int (blue arrow), and sIgM lo (green arrow). **b** Deuterium enrichment curves of sorted CLL cells: sIgMhi (red circles), sIgM int (blue squares), sIgMlo (green triangles). Results are shown from 6 subjects (K4, K6, K7, K8, K9 and K10). **c** Sorting protocol according to expression of BCR within the acidified endosome (eIgM). Gates were set to include 10% of events within each subpopulation: eIgM hi (red arrow), eIgM int (blue arrow), and eIgM lo (green arrow). **d** Deuterium enrichment curves of sorted CLL cells: eIgMhi (red circles), eIgM int (blue squares), and eIgMlo (green triangles). Results are shown from 3 subjects (K6, K9 and K10). For (**b**) and (**d**) data are expressed as fraction of new cells (F %) normalised to one day’s labeling; error bars represent the standard deviation of ≥4 replicate measurements by GC-MS. F Scales vary between subjects to best represent deuterium enrichment. Time represents days post-labeling.

**Fig. 6:**
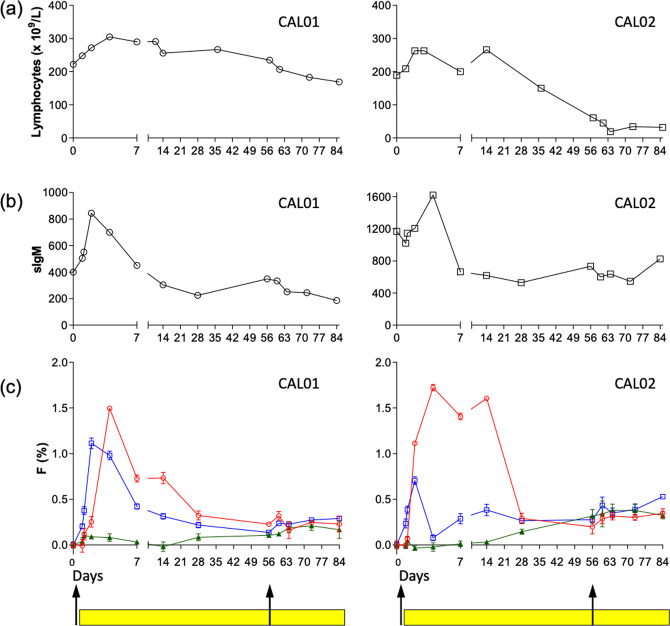
Kinetics of lymphocytosis, median sIgM expression and deuterium enrichment in CLL cells following treatment with idelalisib (CALiBRe study). Results are shown for 2 subjects: CAL01, left panels, and CAL02, right panels, pre- (day 0) and up to 84 days post-treatment with idelalisib: (**a**) Total lymphocyte count; (**b**) Median expression of sIgM; (**c**) Deuterium enrichment in sorted CLL cell subpopulations: sIgM_hi_ (red circles), sIgM_int_ (blue squares), and sIgM_lo_ (green triangles). Data are expressed as fraction of new cells (F %) normalised to one day’s labeling; error bars represent the standard deviation of ≥4 replicate measurements by GC-MS. Black arrows denote oral administration of deuterium labeled glucose. Yellow bars represent administration of idelalisib which was commenced 24 h after the commencement of deuterium labeling. Time points represent days post- first deuterium labeling (day 0).

In vivo labeling was performed by oral administration of 60 g deuterium-glucose (6,6-^2^H_2-_glucose, Cambridge Isotope Laboratories, USA) over 10 h, as previously described [16]. Since labeled glucose disappears rapidly (within hours), this approach labels cells dividing during an approximately 12-hour window. PBMC from follow-up samples (Fig. 2) were cryopreserved in 10% dimethyl sulphoxide prior to sorting (as below), then analysed for DNA deuterium enrichment by gas chromatography-mass spectrometry (GC-MS) as previously described [16, 17]. Results were expressed as the percentage of newly-proliferated cells per day after normalizing for the level and duration of blood glucose labeling directly measured during the labeling phase, as previously described [16] (Figs. 3, 4, 5, 6).

### Immunofluorescence cell sorting

Cryopreserved cells were thawed and stained with fluorochrome-conjugated antibodies to CD5/CD19 either alone or in combination with either CXCR4, sIgM or IgM linked to pHrodo, a dye that fluoresces following internalization into acidic endosomes; for details see Supplementary Table 1. Cells were then flow sorted into fractions representing the whole CLL clone (CD5+/CD19+) or subpopulations defined by expression of CD5 and CXCR4, or IgM at the cell surface (sIgM) or within acidified endosomes (eIgM). Each selected CLL subpopulation comprised 10–15% of the whole CLL cell population, which appeared as a continuum (Figs. 4a, 5a, c). Fluorochromes and gating strategies are detailed in Supplementary Methods.

### Statistical analysis and mathematical modeling

We fitted mathematical models describing the kinetics of CLL cells to the glucose enrichment data. The models assumed that CLL cells undergo proliferation in a compartment “tissue” that is not in immediate dynamic equilibrium with blood, i.e. we assumed that cells neither divide in blood nor in a compartment that freely and immediately recirculates with blood; this assumption precludes a model in which PB1 immediately recirculates with “tissue”. The precise anatomical correlate is immaterial to the modeling. In addition to tissue we included three PB subsets: PB_1_, PB_2_, and PB_3_ representing the three sorted subpopulations: PB_1_: CXCR4_lo_/CD5_hi_, sIgM_hi_ or eIgM_hi_; PB_2_: CXCR4_int_/CD5_int_, sIgM_int_ or eIgM_int_; PB_3_: CXCR4_hi_/CD5_lo_, sIgM_lo_ or eIgM_lo_. Since sIgM and eIgM levels in CLL are closely correlated [14], for the purposes of this study the two were considered together.

Two models of CLL kinetics were considered. In the first (Model A) there are no distinct subpopulations; instead, newly-proliferated CLL cells transit from the tissue compartment, through pools PB_1_, PB_2_ and PB_3_ then back to tissues. In the second (Model B), CLL cells transit from tissues to blood pools PB_1,_ then PB_2_, then back to tissues, whilst the third subpopulation, PB_3_ (CXCR4_hi_/CD5_lo_, sIgM_lo_ or eIgM_lo_ cells), does not re-enter tissues but forms a stable non-proliferating subpopulation in blood. Both models were fitted to deuterium labeling data from bulk and sorted subsets. The small sample corrected Akaike Information Criterion (AICc) was used to determine which model best described the data (based on fit to data penalized by model complexity). Model parameters are fully defined and equations describing the movement between tissue and blood compartments provided in Supplementary Methods.

## Results

### Proliferation, release, and disappearance rates of labeled CLL cells

DNA deuterium labeling curves for bulk tumor cells from this cohort of patients with non-progressive M-CLL are shown in Fig. 3 (for detail of days 0–7 see Supplementary Fig. 2). The curves reflect three processes; (i) the first part of the curve, generally a delay before the appearance of labeled cells, represents the time taken for cells to transit between their site of division and blood, the site of sampling; (ii) the peak primarily reflects the proliferation rate – more proliferation resulting in greater uptake of deuterium during the short labeling phase; (iii) the subsequent disappearance of label from blood primarily reflects the rate at which labeled cells die or migrate out of blood. Analysis of DNA deuterium enrichment curves with these processes in mind led us to four general observations.

Firstly, we noted striking inter-individual heterogeneity in labeling patterns, particularly evident in the release rates of newly proliferated CLL cells into blood. While some subjects such as K8 and K10, demonstrated rapid release of highly-labeled cells, others, such as K5, K6, K9, showed slower release with a lower peak, and some (e.g. K1) showed very little labeling at all. The time to maximum PB deuterium enrichment ranged from 2 to 56 days (median 10.5 days) and was longest in patients with splenomegaly; four of the five patients with very delayed release (≥14 days) had splenomegaly (K4, K5, K6, K9).

Secondly, we observed generally low deuterium labeling rates in tumor cells, consistent with previously reported low proliferation rates in CLL cells [12, 18, 19]. In this study, taking peak labeling as a crude minimum estimate of proliferation (disregarding cell death), we estimated a median proliferation rate of 0.26 %/d (IQR = 0.20–0.32 using cycle 1 data, *n* = 10) equivalent to a median half-life of 271 days (0.29%/d if also including cycle 2 data, *n* = 19).

Thirdly, we found that labeled CLL cells disappeared remarkably slowly (Fig. 3); labeled cells remaining detectable at 56 days in 5/10 cases, suggesting that death rates of labeled cells are low (i.e. that CLL cells have a long lifespan), in keeping with previous observations [6]. Fourthly, although there was wide inter-patient variability, intra-patient reproducibility in repeat studies was generally good (Supplementary Fig. 3).

### CXCR4/CD5 expression predicts proliferative subclonal behaviour and identifies a non-proliferative subpopulation

Analysis of subfractions of PB CLL cells sorted into three non-contiguous populations according to CXCR4 and CD5 expression (Fig. 4) showed that the CXCR4_lo_/CD5_hi_ subfraction appeared in the PB earliest, consistent with previous studies [7]. The intermediate population (CXCR4_int_/CD5_int_) contained less deuterium and took longer to appear in PB, compatible with progressive acquisition of CXCR4 and loss of CD5 expression by the labeled cohort of cells (Fig. 4). The CXCR4_hi_/CD5_lo_ subpopulation, however, exhibited minimal or undetectable labeling throughout the eight-week period of monitoring. Patient K5 proved to have two phenotypically distinct clones and was not included in subsequent analyses.

### Expression of BCR identifies subclonal populations with distinct kinetic characteristics

In view of the previous observation that expression of sIgM is dynamic and that low expression is related to a reversible state of anergy, we went on to investigate proliferation in CLL subsets defined by sIgM expression [5, 13, 20] (Fig. 5a). Assessment of deuterium labeling revealed that CLL cells with the highest sIgM expression (sIgM_hi_) represent the most recently proliferated population with progressively lower labeling in sIgM_int_ and sIgM_lo_ subpopulations (Fig. 5b). Interestingly, in some patients, no detectable label appeared in the sIgM_lo_ cells over the entire two months of the study. We then went on to further investigate the functional relationship between subpopulations defined by the presence of IgM within acidified endosomes (eIgM) in three patients; this measure reflects BCR internalisation and correlates with surface expression [14]. We found that cells with high rates of BCR internalisation (eIgM_hi_) had the highest rates of proliferation whilst the subpopulation with the lowest levels of eIgM remained unlabeled suggesting a fixed state of quiescence (Fig. 5c, d).

### Mathematical modeling frequently predicts the presence of independent subpopulations

We next assessed the relationship between subpopulations using mathematical modeling, testing two alternative hypotheses: (i) that low labeling in the CXCR4_hi_/CD5_lo,_ sIgM_lo,_ eIgM_lo_ populations could be explained by dilution in sequentially labeled pools, or (ii) that CXCR4_hi_/CD5_lo,_ sIgM_lo,_ eIgM_lo_ cells represent a distinct quiescent population. These hypotheses are represented by the two models A and B respectively (Fig. 1), to which the deuterium labeling data was fitted.

Overall, across all data sets (i.e. pooling datasets sorted by CXCR4/CD5 with those sorted by sIgM and eIgM, *n* = 14) Model B provided a substantially better description of the data (difference in AICc >1) in 7 analyses, whereas Model A only outperformed Model B in one case (Table 2). Of these 7 cases, the difference in AICc was large (>=3) for 5 datasets (Table 2). In the other 6 cases, the AICc did not discriminate between the models. Model fits are shown in Supplementary Material. We were therefore able to reject the null hypothesis that Model A always wins with certainty (*p* = 0.008 Wilcoxon signed rank test two sided). This demonstrates that it is unlikely that there is a continuous single population of leukemic cells and that, at least in some patients, recirculation to LNs most likely occurs at the intermediate stage (Model B).

**Table 2:** Comparison of models for CLL subpopulation kinetics.

Trial ID	Sorting	AICc	AICc	Δ AICc	Winning model	Winning model
		Model A	Model B		(*Δ* AICc>1)	large difference
						in AICc
						(*Δ* AICc>3)
K2	CXCR4	−311.6	−317.1	−5.5	B	B
K3	CXCR4	−326.5	−327.3	−0.8	-	-
K4	CXCR4	−351.4	−351.2	0.2	-	-
K5	CXCR4	−290.7	−291.9	−1.2	B	-
K9	CXCR4	−193	−193.9	−0.9	-	-
K10	CXCR4	−187.3	−186.5	0.8	-	-
K4	sIgM	−247.8	−241.2	6.6	A	A
K6	sIgM	−220	−247.6	−27.6	B	B
K7	sIgM	−253.8	−253.1	0.8	-	-
K8	sIgM	−157.2	−157.3	−0.1	-	-
K9	sIgM	−213.9	−219.8	−5.8	B	B
K6	BCR	−212	−214.5	−2.5	B	-
K9	BCR	−231.5	−235.5	−4	B	B
K10	BCR	−196.5	−216.8	−20.3	B	B
				A wins	1	1
				B wins	7	5

Deuterium labeling data in CLL subpopulations (defined by CXCR4/CD5 expression, sIgM expression and eIgM expression) were fitted to two mathematical models: Model A, a single population of leukemic cells in peripheral blood with cells transitioning between phenotypic sub-groups, and Model B, with an independent subpopulation of non-proliferating cells.

Lower AIC_C_ (I.e. more negative) indicate a better fit to the data. The ‘winning’ model is defined by a difference in the AICc of at least 1. Differences of <1 are denoted ‘-‘. The null hypothesis that model A is correct can be rejected with certainty (*p* = 0.008; Wilcoxon signed rank test two sided).

### Idelalisib has a heterogeneous effect on functional subclones

To address the effect of BCR inhibition on these functionally heterogeneous subpopulations, we performed in vivo labeling in two patients (CAL01 and CAL02) with symptomatic CLL participating in the CALiBRe clinical trial. Both had mutated *IGHV* genes and CD38 expression rates >7% (Table 1). Total lymphocyte count and expression of sIgM by CLL cells was recorded over time to assess the kinetics and phenotype of the leukemic clone following drug administration. As anticipated, there was a rapid rise in the lymphocyte count in the days following treatment initiation, peaking on approximately day 4 and falling thereafter (Fig. 6a). This correlated with an abrupt rise in sIgM expression, again maximal on day 2–4 before returning to baseline by day 7, falling to a mean of 43% of peak values by day 56 (Fig. 6b).

Labeling with deuterium-glucose started 24 h before the commencement of idelalisib and was repeated eight-weeks later. The first cycle of pre-treatment labeling was planned to label all cells actively dividing in situ in the absence of drug; idelalisib treatment would not therefore be expected to affect labeling rates in tissue as any deuterium-labeled glucose would have cleared well before idelalisib administration. According to the previous experiments in untreated patients above, such labeled cells would normally remain at their sites of replication for several days before sequential release into blood, resulting in a peak in PB labeling at ~7–14 days (Fig. 4). By contrast, both study patients showed a prompt increase in labeling rates in PB CLL cells, apparent within four hours of idelalisib dosing (Fig. 6 and in detail for days 0–7 in Supplementary Fig. 4). Labeling peaked on day 4 and was greatest in the sIgM_hi_ compartment; by contrast, labeling in the sIgM_lo_ compartment remained undetectable indicating that sIgMhi cells represent the most recently proliferated population.

Strikingly, when rates of labeling were re-evaluated eight weeks into idelalisib treatment, there was a dramatic, near-complete cessation of proliferation in all leukemic cell subsets (Fig. 6c), evident as the absence of an expected second peak in deuterium enrichment. In untreated individuals this peak occurred predictably 0–7 days after labeling (Fig. 5b), especially in the sIgM_hi_ subpopulation and, to a lesser extent, in sIgM_int_ cells; for the second (d56) labeling, this peak would be expected at 56–63 days post-study initiation. Comparing the red lines at d0-7 in Fig. 5b with those at d56-63 in Fig. 6c, the extent to which proliferation appears to be blocked can be appreciated. At this time-point, both treated patients still had a persisting lymphocytosis but median sIgM expression levels were reduced compared to pre-treatment levels and labeling rates were now virtually undetectable in all sorted subpopulations suggesting that CLL cells expressing high levels of sIgM are most sensitive to BCR-directed treatment.

## Discussion

In this study, we investigated whether the leukemic cells of CLL patients are homogeneous in their capacity to undergo proliferation or contain distinct subpopulations that differ in this respect. Our results convincingly show that CLL does not always behave as though the tumor is functionally homogeneous and that, at least in this population of patients with mutated *IGHV*, distinct subsets of cells with different proliferative capacities can be identified using phenotypic markers.

Uniquely, our study used in vivo pulse-chase labeling with deuterated-glucose to investigate this important question. To increase our capacity to detect non-proliferating subpopulations, we focussed on patients with mutated *IGHV* who are already known to have lower proliferation rates. As expected, we found very low estimated rates of in vivo tumor proliferation (median 0.26%/day), consistent with previous reports with deuterated glucose (0.57%/d) [12] or deuterium-labeled water (0.36%/d) [19], albeit in different patient populations. The delay to peak appearance of newly-produced leukemic cells in blood is consistent with cell division in the non-blood compartment for all subclones. This lag was highly variable and longest in the four patients with marked splenomegaly, suggesting that spleen and blood together form recirculating pool; when this is large, as in splenomegaly, labeling is slow. In keeping with previous studies, we found that the rates of disappearance of CLL cells from the peripheral blood were much slower than those reported for healthy B lymphocytes [12]. This finding might result from impaired apoptosis in CLL cells [12], but could also reflect trapping of CLL cells in blood.

Analysis of the deuterium content of flow-sorted subpopulations clearly revealed sequential incorporation into CXCR4_lo_/CD5_hi_, then CXCR4_int_/CD5_int_, then CXCR4_hi_/CD5_lo_ fractions, consistent with a model in which CXCR4_lo_/CD5_hi_ cells represent the actively-dividing population. (The alternative hypothesis that all subpopulations divide but appear sequentially because they have different release dynamics is refuted by the observation that CXCR4_lo_/CD5_hi_ cells comprise the population enriched for proliferating cells within the LN compartment [8]). However, our observations that deuterium enrichments were substantially lower in CXCR4_int/_CD5_int_ cells, and even lower (occasionally undetectable) in the CXCR4_hi_/CD5l_o_ fraction, suggests that exchange of deuterium between phenotypic groups over time may be limited, in contrast to the conclusions of previous work using deuterated water [7].

Similar patterns were observed when cells were sorted according to sIgM or eIgM levels, both markers of BCR signaling capacity. Deuterium labeling was greatest and most rapid in the sIgM_hi/_ eIgM_hi_ fraction, consistent with high sIgM expression in LNs, the sites of antigen encounter and proliferation [14, 21]. (Although it may seem counterintuitive that cells remain sIgM_hi_ despite recent BCR ligation, levels may be maintained by microenvironment-related factors such as CD4 T-cell derived IL4 which enhances sIgM expression and BCR signaling whilst inducing reciprocal downregulation of CXCR4 [22, 23].) The most striking finding, however, was that, as for CXCR4/CD5 sorting, a subpopulation defined by reduced surface or endosomal BCR expression remained almost entirely unlabeled throughout the period of the study.

When these patterns of labeling of CLL subpopulations were analysed using mathematical modeling we found very little support for sequential labeling model (Model A) and refuted the null hypothesis that ‘Model A is true in all cases of M-CLL’. Conversely, our data suggest that, at least in some if not all patients, most CLL cells return to LN as CXCR4_int/_CD5_int_/sIgM_int_, eIgM_int_ cells, to re-emerge after proliferation as CXCR4_lo_/CD5_hi_/sIgM_hi/_eIgM_hi_ cells. Meanwhile, the CXCR4_hi_/CD5l_o_/sIgM_lo_/eIgM_lo_ subpopulation forms a quiescent long-lived pool of cells that either does not migrate and proliferate, or if it does, only at rate lower than the limit of detection of the method described, a possibility which is perhaps more likely as some input of new cells is required to balance cell death and preserve population size. In terms of phenotypic characterisation, previous studies have shown that the CXCR4_hi_/CD5l_o_ population is enriched for CCR7 and CTLA4 expression, in contrast to the CXCR4lo/CD5 fraction which is enriched for CD38 and CD62L [7].

**Fig. 7:**
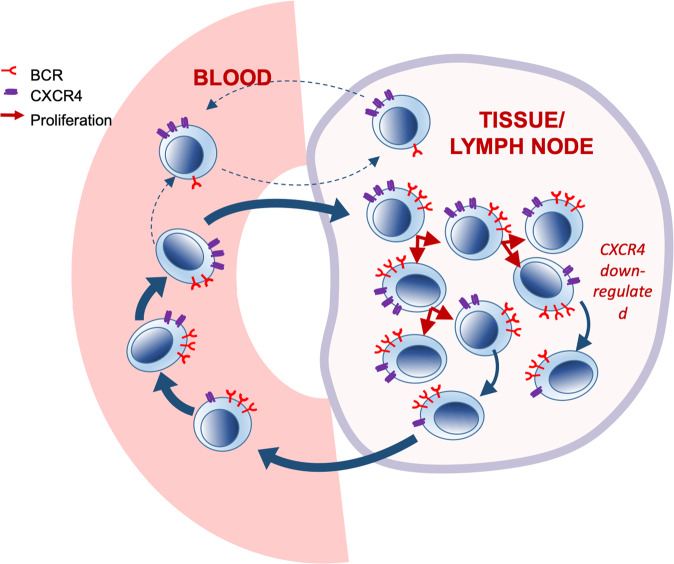
Hypothetical model of leukemic cell kinetics in untreated *IGHV* mutated CLL. Proposed model of tumor kinetics in CLL that fits the deuterium labeling data in the present study. A proliferative subset of cells transits between PB and lymphoid tissues. These cells become labeled with deuterium, change their phenotype from CXCR4_lo_CD5_hi_ to CXCR4_int_CD5_int_ and express high levels of surface BCR. A largely distinct non-proliferating subset is also present. This subset does not label with deuterium, has a CXCR4_hi_CD5_lo_ phenotype and low expression of surface BCR. These cells generally do not migrate into lymphoid tissues but, unlike normal anergic B cells, which undergo apoptosis when excluded from germinal centres, these cells express bcl-2 and so survive.

A hypothetical model, building upon previously-published models [7] is shown in Fig. 7. This interpretation is consistent with work performed using an in vitro circulation system showing that the leukemic clone has heterogeneous kinetics and that migration is limited to a small sub-population characterised by an activated lymph-node phenotype [10, 24]. It is also consistent with recent studies showing that subclonal diversity correlates with the in vivo growth characteristics of CLL [11]. Interestingly, in their study, logistic tumor growth pattern, with plateauing of cell numbers after an initial increase, was observed in patients with lower subclonal complexity and mutated *IGHV*, and it is plausible that this may be a consequence of the emergence of the non-proliferating subset described herein.

Finally, although necessarily limited to two patients by regulatory issues beyond our control, pre-treatment deuterium labeling showed that idelalisib therapy induces rapid release of recently-proliferated sIgM_hi_ cells into PB, consistent with the observation that Ki67 [25] and sIgM expression [26] in circulating CLL cells rapidly rises following initiation of treatment with ibrutinib, likely as a consequence of release from lymphoid tissues. After eight weeks of treatment with idelalisib, deuterium labeling data showed cessation of proliferation of the sIgM_hi_ subpopulation, as expected from reports that CLL cells expressing high levels of sIgM (the main determinant of BCR signaling) are most sensitive to BCR inhibition [13, 20]. The quiescent sIgM_lo_ population meanwhile persisted, perhaps because it is less dependent on BCR signaling and so less sensitive to its inhibition (Supplementary Fig. 6). Selection of the non-proliferating CXCR4_hi_CD5_lo_, sIgM_lo_ subset may explain the persistent low-level disease observed during long term ibrutinib therapy [9]. This idea is supported by changes in the tumor immunophenotype over time during ibrutinib therapy as sIgM, eIgM and CD5 expression levels initially increase then fall in the longer term [14, 26].

In terms of limitations, although we studied only 10 + 2 subjects, our conclusions are supported by replicate measurements for every sample/time-point and repeated measurements over time within subjects, giving an overall database of over 500 enrichment measurements (Supplementary Material). Inter-individual variation was marked, as would be expected in such a heterogenous disease, and the overall pattern of labelling varied widely between patients (Fig. 3), but despite this the hierarchy and sequential nature of labeling in defined subpopulations was remarkably similar between patients (Fig. 4 & 5). Our primary finding regarding model comparisons is highly unlikely to have arisen by chance (*p* = 0.008).

Our results raise important further questions. Firstly, how do these functionally distinct subsets arise? The link between anergy and lack of capacity to migrate into LN follicles has been previously described in normal murine B-cells [27]. Our hypothesis is that the non-migratory/non-proliferating CLL subset we have identified resembles anergic normal B-cells in that both are excluded from entry into follicles but, whereas the latter undergo apoptosis, CLL cells are rescued by *bcl-2* expression. Detailed genetic and biochemical studies were outside the scope of the present study, but would determine whether the previously identified clonal heterogeneity [1, 11, 28] is linked to the functional and kinetic differences we have described here. Secondly, if there is a non-proliferative component in some cases of CLL, is it necessary to eliminate every detectable leukemic cell to obtain lasting clinical benefit? Many patients with monoclonal B-lymphocytosis or stage A CLL remain well for prolonged periods; if it is possible to eliminate the more dangerous proliferative component of the disease with drugs such as ibrutinib, then perhaps therapy can be discontinued once only non-proliferative/anergic cells remain. The observation of prolonged disease stability in some patients who have discontinued ibrutinib because of side effects suggests that this may be the case [29].

## Supplementary information

Supplemental Material
