# Supplementary material for: Identification of proliferative and non-proliferative subpopulations of leukemic cells in CLL
Source: Leukemia. 2022 Jul 28;36(9):2233–41. doi: 10.1038/s41375-022-01656-4 (PMC9417999; doi:10.1038/s41375-022-01656-4)
Supplement: Supplementary file 1 — Supplemental Material [file 41375_2022_1656_MOESM1_ESM.pdf]

Supplementary Material

Supplementary Methods 1: Immunofluorescence Cell Sorting

Supplementary Table 1. Antibodies used for Flow Cytometry

Supplementary Methods 2: Mathematical Modeling.

Supplementary Figure 1. Parameterised models of CLL proliferation and recirculation

Supplementary Results

Reproducibility of GC-MS measurements

Supplementary Table 2. Variance of replicate measures of isotope enrichment

sIgM Expression following idelalisib therapy

Supplementary Table 3. sIgM Expression following idelalisib therapy

Supplementary Figures

Supplementary Figure 2. Detail of first seven days of deuterium labelling in $CD19^+$ leukemic cells

Supplementary Figure 3. Comparison of deuterium enrichment curves in $CD19^+$ leukemic cells after one or two cycles of labeling

Supplementary Figure 4. Detail of first seven days deuterium labeling in leukemic subpopulations with idelalisib administration starting on day one after labeling

Supplementary Figure 5. Time-course of lymphocytosis following idelalisib dosing

Supplementary Figure 6. Hypothetical model for leukemic cell dynamics following BCR-inhibitor therapy

Supplementary References

Plots of Final fits

Supplementary Methods 1: Immunofluorescence Cell Sorting

Cryopreserved cells were thawed and stained with fluorochrome conjugated antibodies to CD5/CD19 either alone or in combination with CXCR4, IgM or IgM linked to pHrodo, a dye that fluoresces following internalization into acidic endosomes (Table S1).

Measurement of IgM expression within acidified endosomes was performed by incubating one microgram of pHrodo™ Red Avidin with or without an equimolar amount of biotinylated goat $F(ab')_2$ anti-human immunoglobulin M (αIgM; Cambridge Biosciences, Cambridge, UK) for one hour at room temperature to allow formation of labeled complexes. Target cells (500, 000) were incubated with pHrodo-avidin-αIgM for 30 minutes at 4°C to allow receptor binding, then at 37°C for a further 60 minutes to allow for internalization. All incubations were performed in PBS at pH 7.4 so that any observed immunofluorescence was due to BCR internalization and trafficking of the pH rodo-αIgM into acidified endosomes, BCR internalization was expressed as the percentage of cells with fluorescence above that of the pHrodo-avidin negative control.[1]

Labeling was performed on ice and CLL cells were washed twice with PBS. DAPI was used to label non-viable cells.

Cells were then flow-sorted (BD FACSAria™ II Cell Sorter) into fractions representing the whole CLL clone (CD5+/CD19+) or subpopulations defined by the levels of expression of CD5 and CXCR4, or IgM at the cell surface (sIgM) or within acidified endosomes (eIgM).

<u>CD5/CXCR4 Sort:</u> Viable CLL cells were sorted into cohorts according to expression of CXCR4 and CD5, taking the 15% (minimum 500, 000) cells with highest CXCR4 and lowest CD5 expression ($CXCR4_{hi/}CD5_{lo}$), the 15% cells with lowest CXCR4 and highest CD5 expression ($CXCR4_{lo}/CD5_{hi}$)and the intermediate 15% cells ($CXCR4_{int}/CD5_{int}$). The gating strategy is illustrated in Figure 4.

<u>sIgM Sort:</u> Viable cells were sorted into three cohorts according to expression of sIgM, taking the 10% cells with the highest sIgM expression ($sIgM_{hi}$),the 10% cells with lowest sIgM expression ($sIgMl_o$) and the 10% cells with intermediate sIgM expression ($sIgM_{int}$).

<u>eIgM Sort:</u> Viable cells were sorted into cohorts taking the 10% cells with highest ($eIgM_{hi}$), intermediate ($eIgM_{int}$) and lowest ($eIgM_{lo}$) eIgM expression.

Sorted subfractions were stored as cell pellets at -80 °C prior to analysis of DNA deuterium enrichment by gas chromatography-mass spectrometry (GC-MS). Details of fluorochromes are provided in Table S1.

Supplementary Table 1. Antibodies used for Flow Cytometry

Antibody	Clone	Supplier
CD5 – FITC	UCHT2	Biolegend
CD5 - PECy7	UCHT2	Biolegend
CD19 – APC	HIB19	Biolegend
CD19 – PB	HIB19	Biolegend
CXCR4 – PE	12GS	Biolegend
Anti-IgM	FITC	Biolegend
pHrodo™ Red Avidin		Life Technologies, Paisley, UK

Supplementary Methods 2: Mathematical Modeling.

Two mathematical models encapsulating two alternative hypotheses were constructed (Supplementary Figure 1).

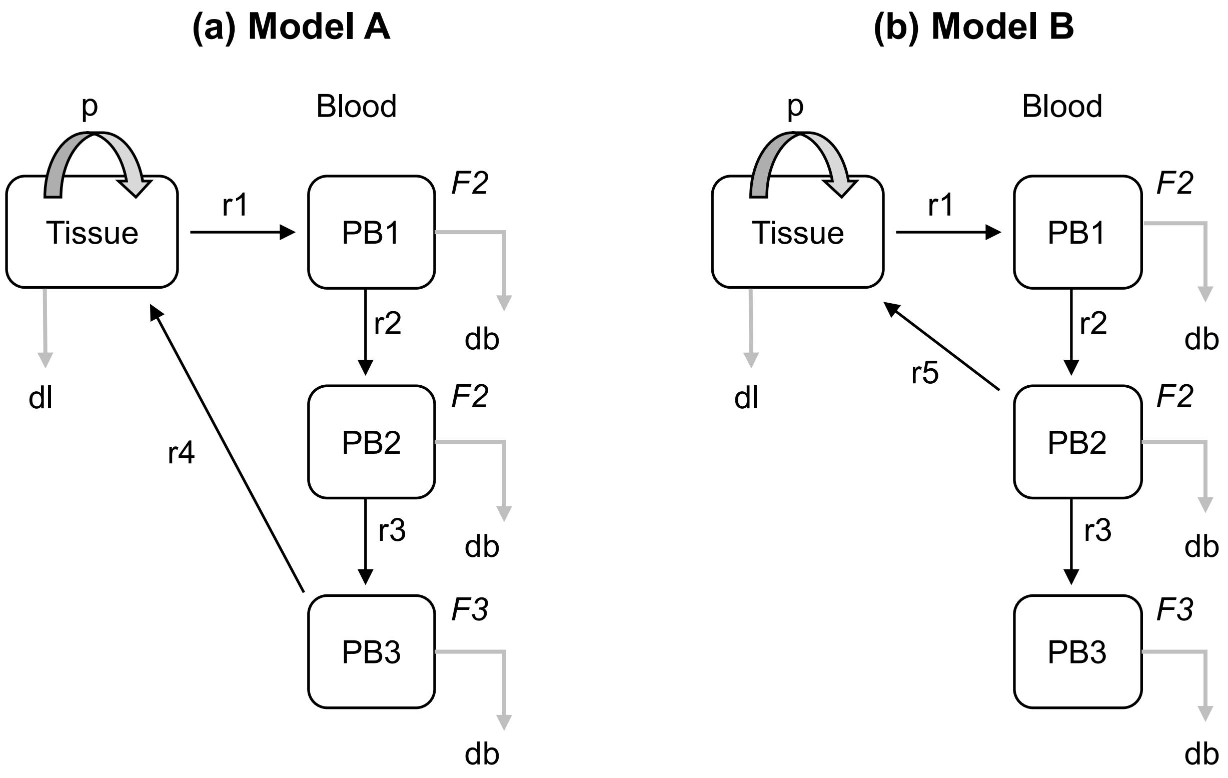

Supplementary Figure 1. Parameterised models of CLL proliferation and recirculation

Tissue represents the non-blood sites of cell proliferation at rate p; solid arrows represent transitions between subpopulations denoted by rates, r1 … r5. Cell disappearance (primarily death) is represented by gray arrows with rates of dl, from the non-blood compartment, and db, from the blood compartment. The three blood phenotypes are defined as follows:

PB1: $CXCR4_{lo}/CD5_{hi}$, $sIgM_{hi}$ or $eIgM_{hi}$;

PB2: $CXCR4_{int}/CD5_{int}$, $sIgM_{int}$ or $eIgM_{int}$, and

PB3: $CXCR4_{hi/}CD5_{lo}$, $sIgM_{lo}$ or $eIgM_{lo}$, respectively.

These correspond to the following equations:

- For Model A:

$$\begin{aligned}
\frac{dL}{dt} &= pL - (r_1 + d_l)L + r_4 PB_3 \\
\frac{dPB_1}{dt} &= r_1 L - (r_2 + d_b)PB_1 \\
\frac{dPB_2}{dt} &= r_2 PB_1 - (r_3 + d_b)PB_2 \\
\frac{dPB_3}{dt} &= r_3 PB_2 - (r_4 + d_b)PB_3
\end{aligned} \tag{1}$$

- For Model B:

$$\begin{aligned}
\frac{dL}{dt} &= pL - (r_1 + d_l)L + r_5 PB_2 \\
\frac{dPB_1}{dt} &= r_1 L - (r_2 + d_b)PB_1 \\
\frac{dPB_2}{dt} &= r_2 PB_1 - (r_3 + r_5 + d_b)PB_2 \\
\frac{dPB_3}{dt} &= r_3 PB_2 - d_b PB_3
\end{aligned} \tag{2}$$

Where L is the number of CLL cells in the tissue and PB_x is the number of CLL cells in the PB_x compartment. The CLL cells proliferate with rate p, die with rate d_l in the tissue, die with rate d_b in the blood and move between compartments with rates r_i . (In Model B, we experimented with a different death rate d_{b3} in the PB_3 compartment but this never resulted in improved fits.) We assume all of these populations are at steady state for the duration of the experiment.

The equations describing the fraction of label in the different compartments are:

- Model A:

$$\begin{aligned}
\frac{dF_L}{dt} &= p\left(Ub - F_3\right) + d_l\left(F_3 - F_L\right) + r_1 F_3 - r_1^* F_L \\
\frac{dF_1}{dt} &= \left(r_2 + d_b\right)\left(\frac{r_1^*}{r_1} F_L - F_1\right) \\
\frac{dF_2}{dt} &= \left(r_3 + d_b\right)\left(F_1 - F_2\right) \\
\frac{dF_3}{dt} &= \left(r_4 + d_b\right)\left(F_2 - F_3\right)
\end{aligned} \qquad (3)$$

- Model B

$$\begin{aligned}
\frac{dF_L}{dt} &= p\left(Ub - F_2\right) + d_l\left(F_2 - F_L\right) + r_1 F_2 - r_1^* F_L \\
\frac{dF_1}{dt} &= \left(r_2 + d_b\right)\left(\frac{r_1^*}{r_1} F_L - F_1\right) \\
\frac{dF_2}{dt} &= \left(r_3 + d_b + r_5\right)\left(F_1 - F_2\right) \\
\frac{dF_3}{dt} &= d_b\left(F_2 - F_3\right)
\end{aligned} \qquad (4)$$

Where F_L is the fraction of label in cells in tissue, while F_x is the fraction of label in the PB_x compartment; $U(t)$ is the precursor enrichment (modelled as a plateau during deuterium administration with an exponential decay afterwards) and b = 0.73. We have allowed a different exit rate r_1^* for cells that have recently divided in the lymph compartment. During the fitting process we restricted the relative sizes of the blood compartments, as each of them has been represented with a sample that is at least 10% of the total CLL blood compartment. Furthermore, we restricted the size of the CLL population in the tissue to be at least as big as the size of the CLL population in the blood compartment.

The two models given by (3) and (4) were fitted to the experimental data (observables: label in each of the three subpopulations F_1, F_2, F_3 and label in the total population (F1 PB1+F_2 PB_2+F3 PB3)/(PB_1+PB_2+PB_3) by minimizing the sum of squared residuals using the pseudo Optim algorithm from the FME package in R [2,3]. Model A has 8 parameters: p, d_l, d_b, r_1, r_2, r_3, r_4, r_1*; three population ratios L/PB1, PB1/PB2, PB2/PB3 and, (by solving equations (1) for

equilibrium), four equilibrium constraints giving a total of 8+3-4=7 free values to be fitted; Model B has 9 parameters: p, d_l, d_b, r_1, r_2, r_3, r_4, r_5, r_1* and, as for model A, three population ratios and four equilibrium constraints (obtained by solving equations (4) for equilibrium) giving a total of 8 free values to be fitted. The number of data points per person varied between individuals from 28-40 (mean 32). Fits were compared using the small sample corrected Akaike Information Criterion (AICc)[4].

Supplementary Results

Reproducibility of GC-MS measurements

Each sample was analysed at least in quadruplicate after having been abundance-matched by dilution or concentration so as to eliminate artefactual effects of sample size on measured isotope ratios. The standard deviations of each set of replicate measurements were collated according to cell sorting strategy (Table S2).

Supplementary Table 2. Variance of replicate measures of isotope enrichment

Cell sorting strategy	n	Median SD
CD19	166	0.029
CXCR4/CD5	183	0.031
sIgM	117	0.036
eIgM	54	0.034

N is the number of independent samples analysed in each group. The median SD is expressed in the same units as the enrichment data, i.e. normalised to the fraction of new cells equivalent to a one-day labeling period (F, %/day) by dividing by the area under curve of the measured glucose enrichment data during labeling.

In terms of minimal detectable enrichments. taking a typical SD of 0.03 gives a 90% chance of detecting a difference equivalent to 0.07 %/d between two samples with n=4 measurements for each sample. Since multiple measurements are made along the labeling curve, the minimal detectable proliferation rate in CLL cells is likely to be less than this figure. Typical peak enrichments in this study are of the order of 0.20-0.32 %/day equivalent (1st to 3rd IQR).

Supplementary Table 3. sIgM Expression following idelalisib therapy

Time (days)	CAL01	CAL02
0	400	1167
1	504	1020
1.2	551	1144
2	844	1205
4	700	1619
7	451	664
14	304	618
28	225	529
56	349	733
60	334	600
64	251	637
72	244	544
84	186	824

sIgM expression (median fluorescent intensity measured using flow cytometry) by CLL cells was measured at regular intervals between Day 0 and Day 84 post idelalisib dosing.

Supplementary Figures

Supplementary Figure 2. Detail of first seven days of deuterium labelling in CD19$^+$ leukemic cells

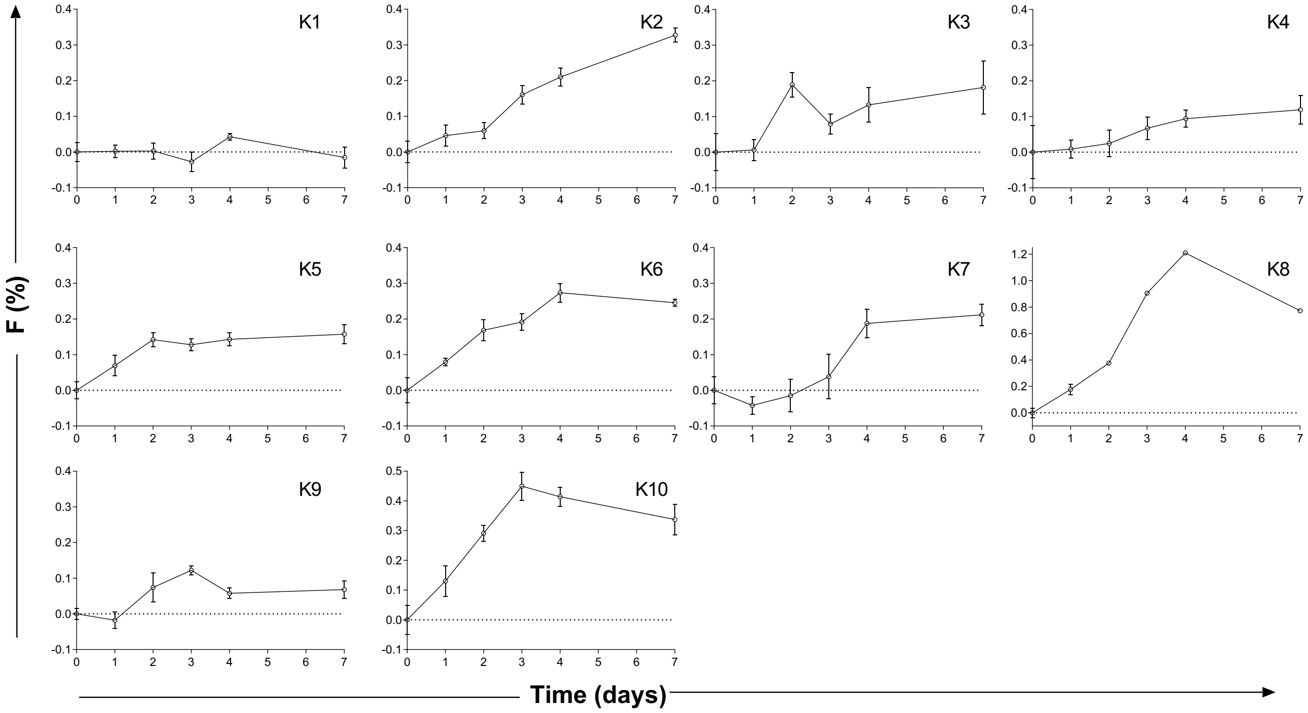

Data as in Figure 3 but only for days 0-7.

Supplementary Figure 3. Comparison of deuterium enrichment curves in CD19⁺ leukemic cells after one or two cycles of labeling

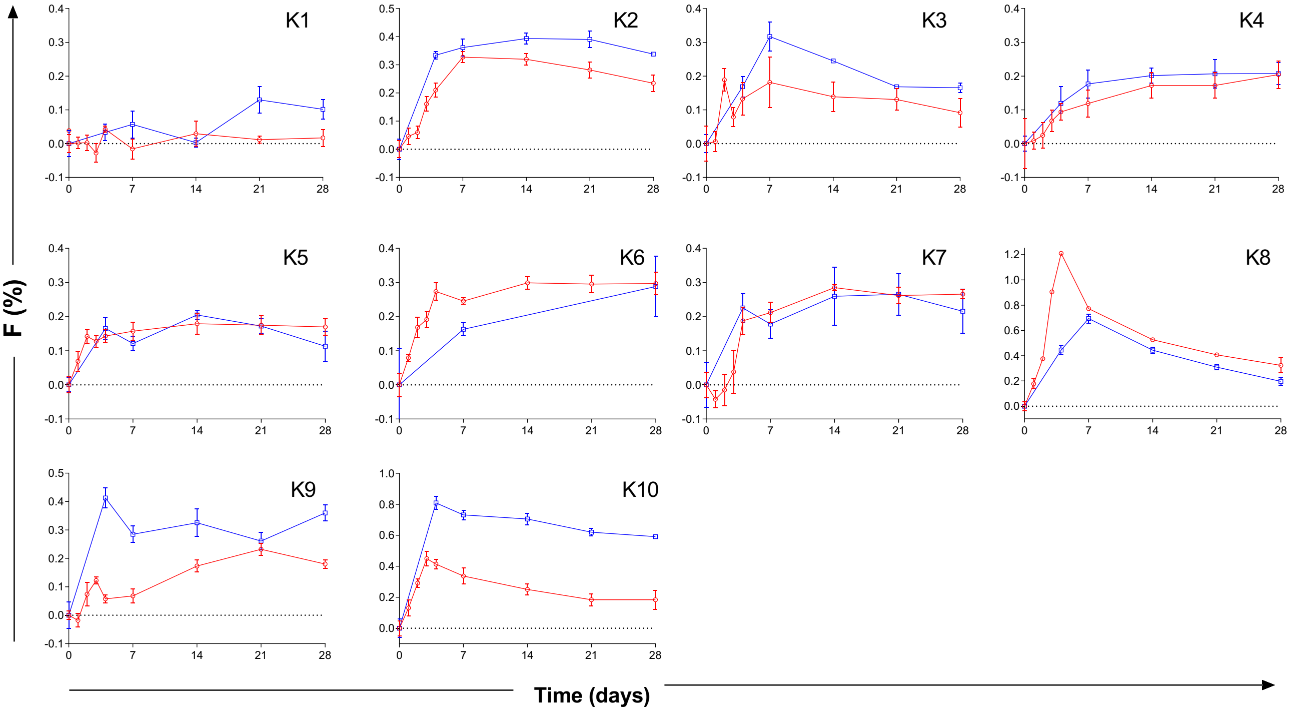

Figures show deuterium labeling rates as fraction of new cells (F), normalised to a one-day labeling phase, for flow-sorted CLL cells after one (red) or two (blue) cycles of labeling 8 weeks apart. Cycle 2 values are corrected by subtracting the residual enrichment at day 56 of the first cycle. Variation between cycle 1 and 2 in K10 may reflect an underestimate of residual labeling at the new baseline for cycle 2 (day 56 of cycle 1). Time represents days post-labeling.

Supplementary Figure 4. Detail of first seven days deuterium labeling in leukemic subpopulations with idelalisib administration starting on day one after labeling

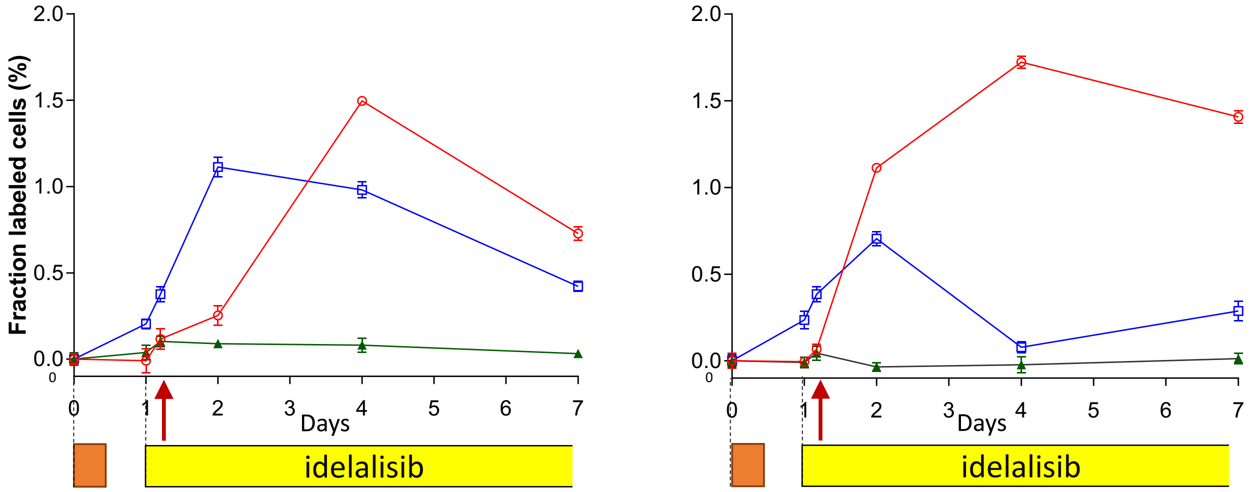

Data as for Figure 6 but only for days 0-7. Deuterium enrichment is shown for sorted CLL cell subpopulations: sIgM$_{hi}$ (red circles), sIgM$_{int}$ (blue squares), and sIgM$_{lo}$ (green triangles). Orange block marks the administration time of deuterated glucose on day 0. Red arrow denotes 4-hours post-idelalisib dose blood sampling.

Supplementary Figure 5. Time-course of lymphocytosis following idelalisib dosing

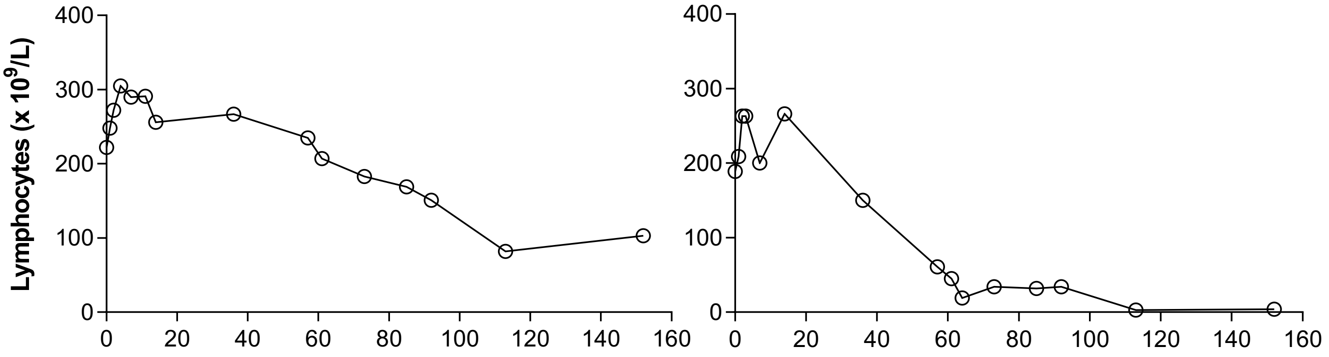

Peripheral blood lymphocyte count was measured at regular intervals between Day 0 and Day 120 post dosing with idelalisib. Panels show total lymphocyte count in (a) Patient CAL01 and (b) Patient CAL 02.

Supplementary Figure 6. Hypothetical model for leukemic cell dynamics following BCR-inhibitor therapy

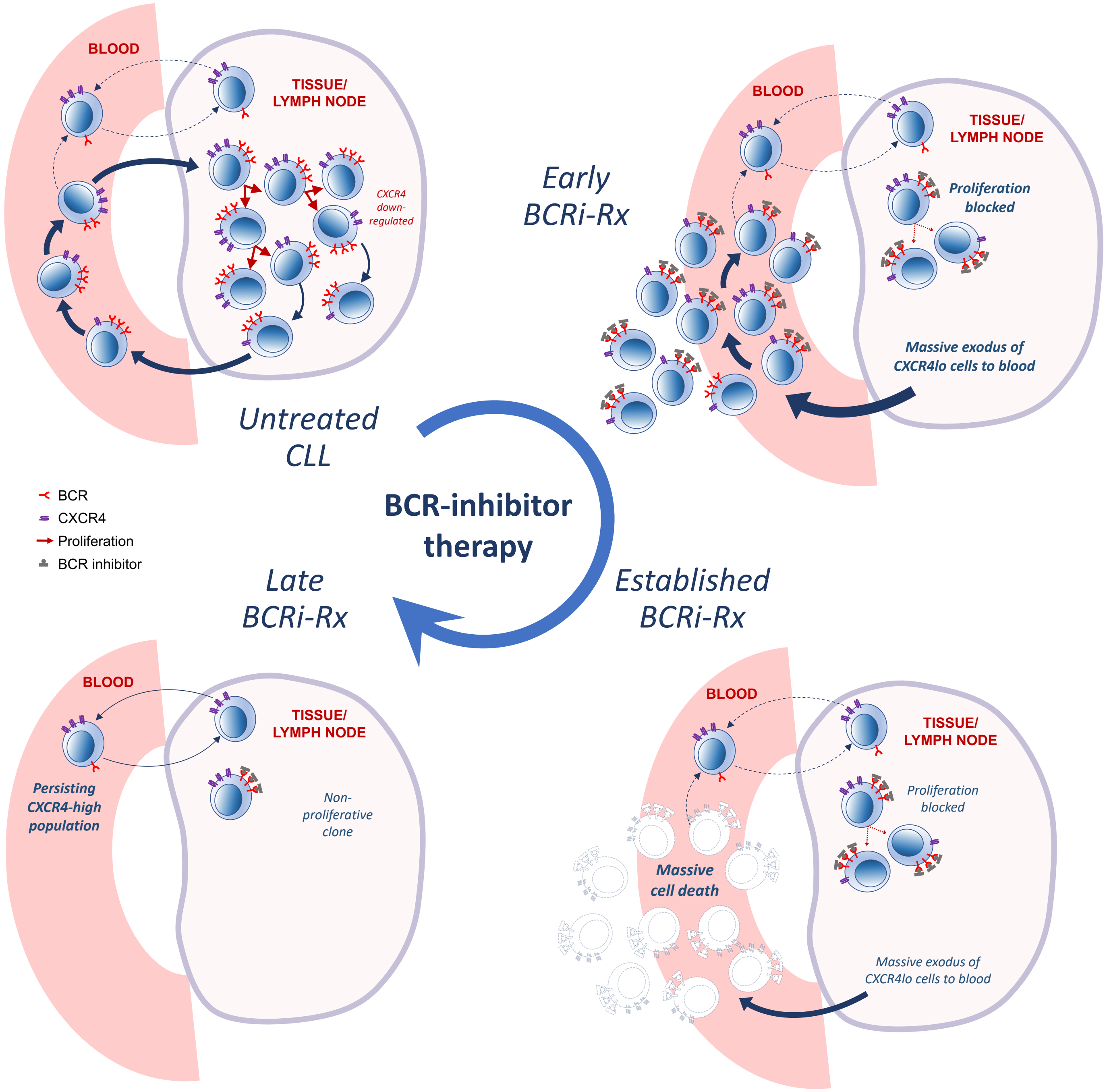

Proposed tumor dynamics in patients receiving idelalisib (or other BCR pathway inhibitors). Initiation of idelalisib results in rapid efflux of recently proliferated $CXCR4_{lo}CD5_{hi}/sIgM_{hi}$ into the peripheral blood. These cells die as a result of BCR pathway inhibition and absence of trophic signals from the microenvironment. A population of non-proliferating, mainly non-migratory peripheral blood $CXCR4_{hi}CD5_{lo}/sIgM_{lo}$ tumor cells remains. This population is less dependent on BCR signaling and the tissue microenvironment and is selected for by BCR pathway inhibition.

Supplementary References

1. Coulter EM, Pepper A, Mele S, et al. In vitroand in vivoevidence for uncoupling of B-cell receptor internalization and signaling in chronic lymphocytic leukemia. *Haematologica*. 2018;103(3):497–505.

2. Soetaert, K. R Package FME : Inverse Modelling, Sensitivity,Monte Carlo – Applied to a Dynamic Simulation Model. URL https://cran.r-project.org/web/packages/FME/.

3. R: A language and environment for statistical computing. URL http://www.R-project.org/. (2014).

4. Burnham, K.P. & Anderson, D.R. *Model selection and multimodel inference*. Springer-Verlag: New York, NY, 2002.

Final set of model fits for homogenous model (model A) and separate subpopulation model (model B)

		model B	model B fit	model A	Model A fit
Subject	Sorting	via B2		via B3	
K2	CXCR4	-317.12	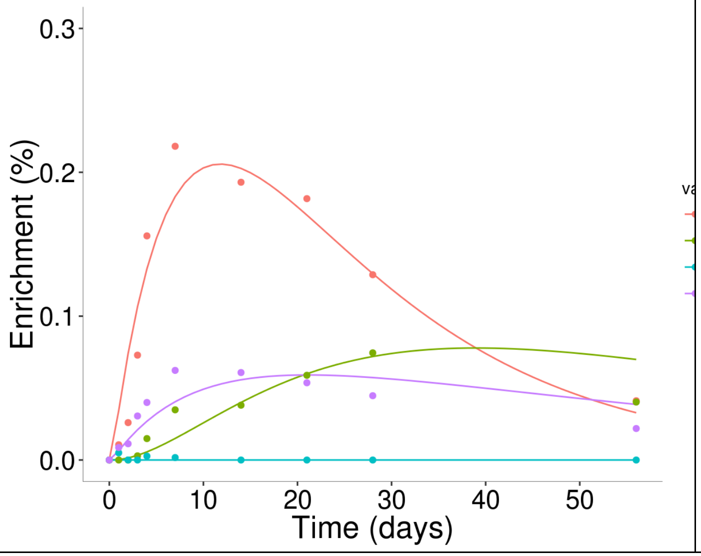	-311.62	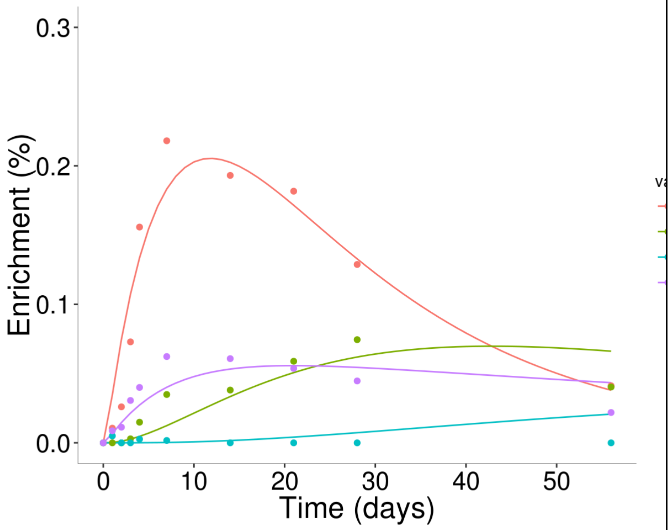

K3	CXCR4	-327.29	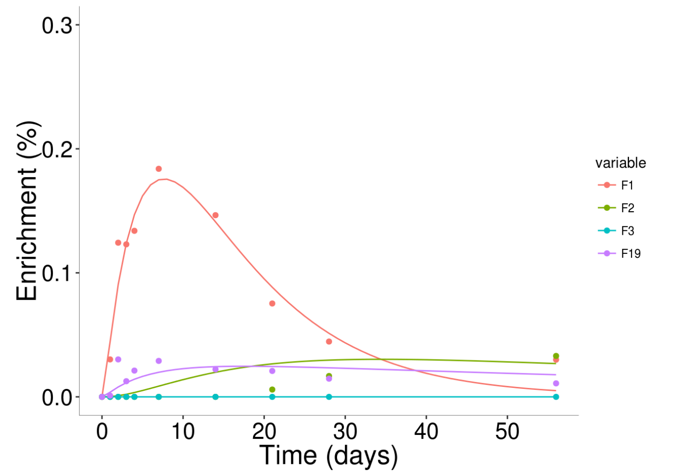	-326.46	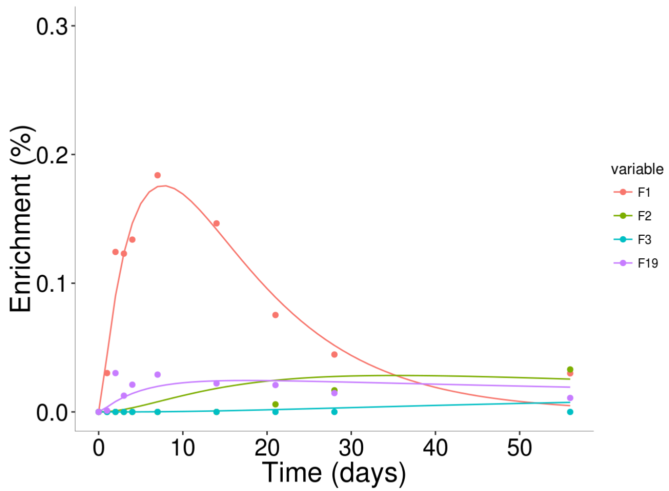
K4	CXCR4	-351.2	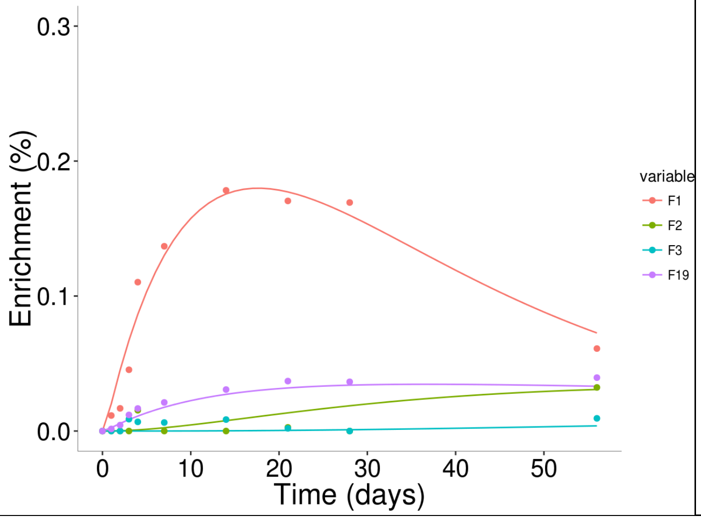	-351.35	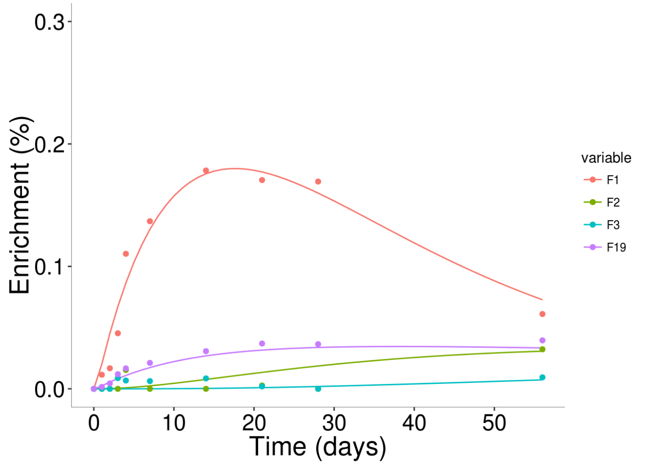

<table>
<tr><td>K5</td><td>CXCR4</td><td>-291.93</td><td>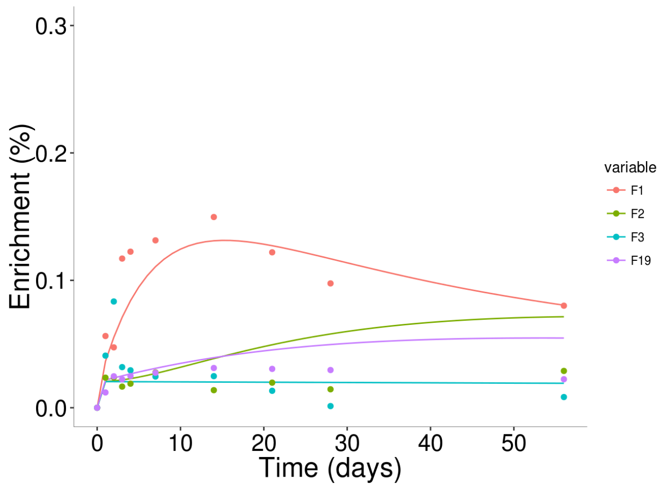
</td><td>-290.71</td><td>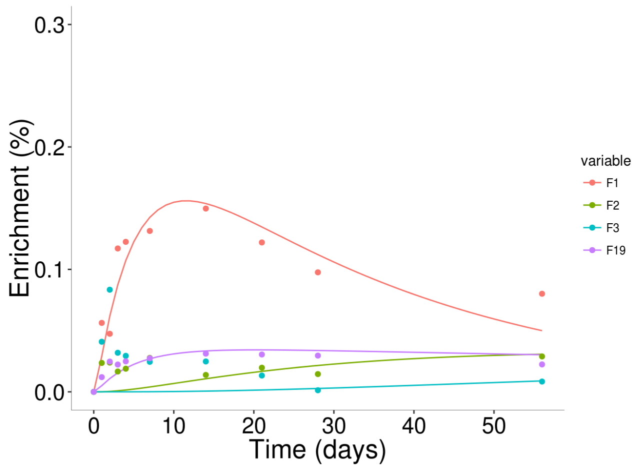
</td></tr>
<tr><td>K9</td><td>CXCR4</td><td>-193.94</td><td>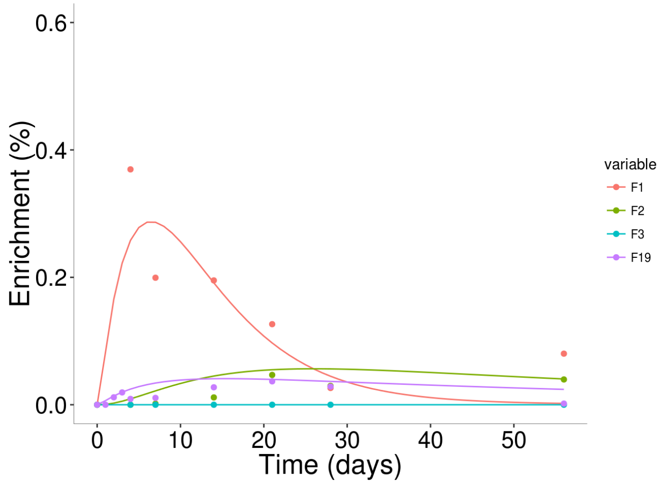
</td><td>-193.02</td><td>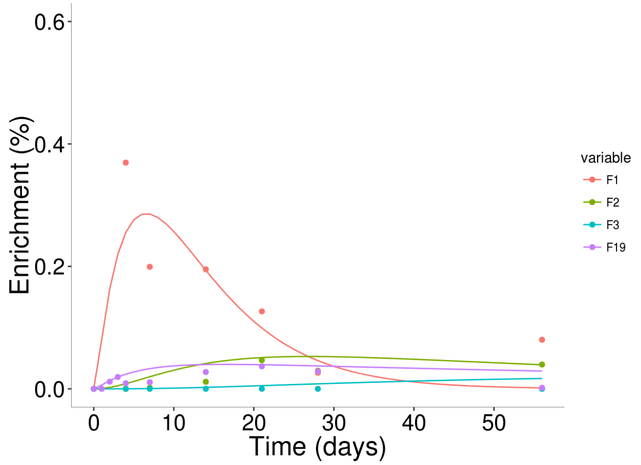
</td></tr>
</table>

K10	CXCR4	-186.51	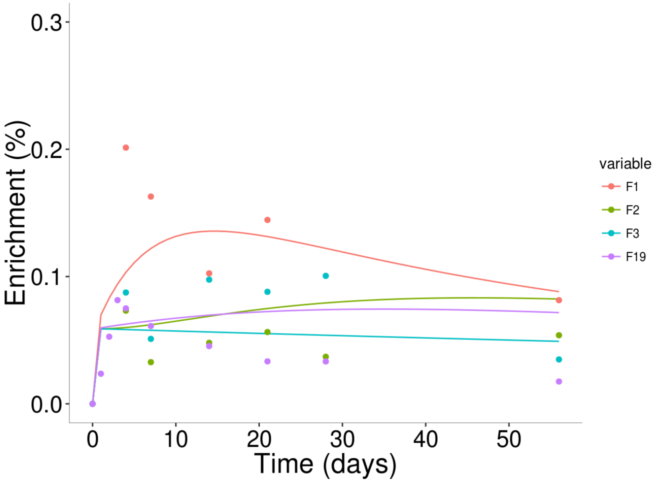	-187.29	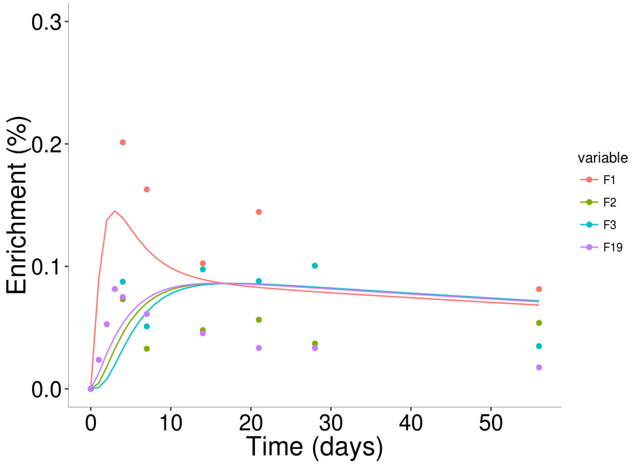
K4	sIgM	-241.23	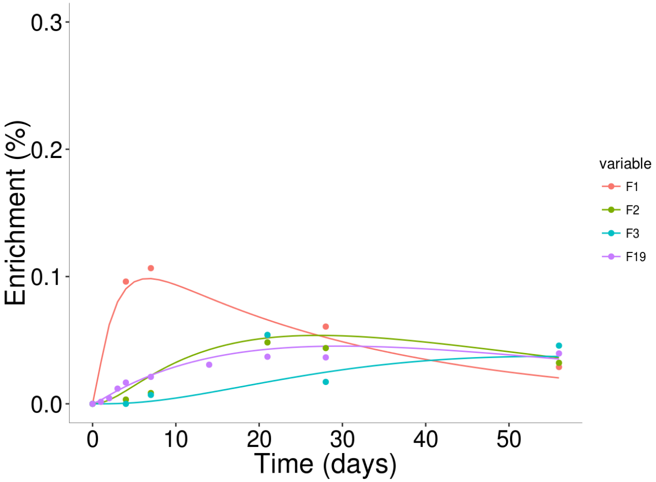	-247.78	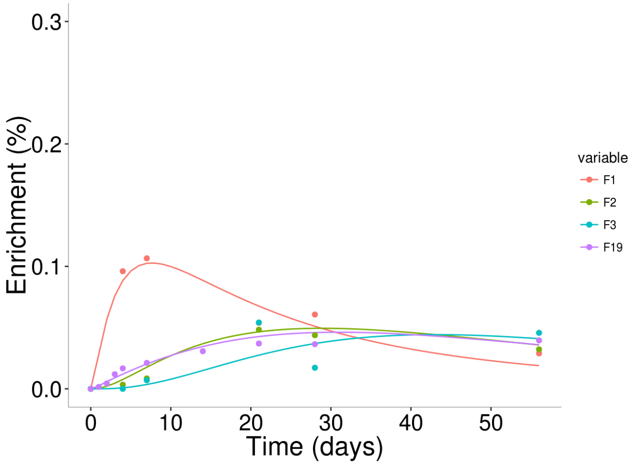

K6	sIgM	-247.63	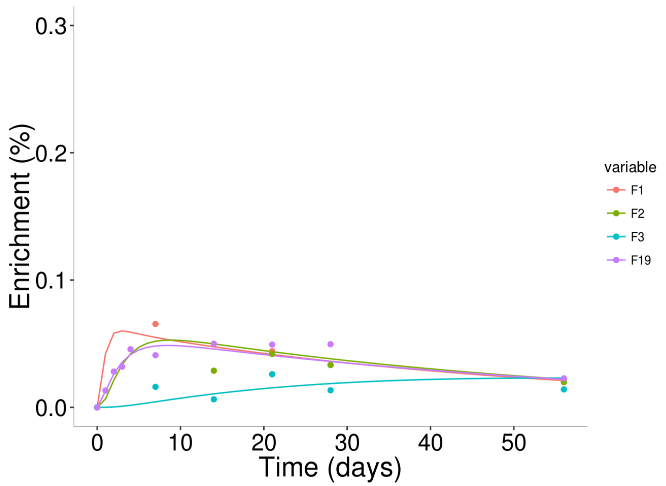	-220	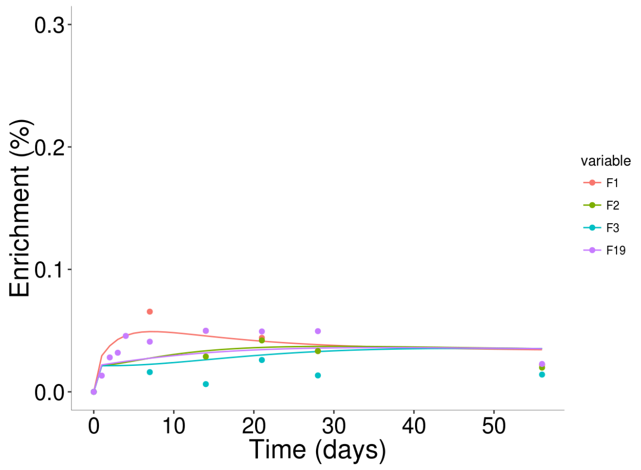
K7	sIgM	-253.05	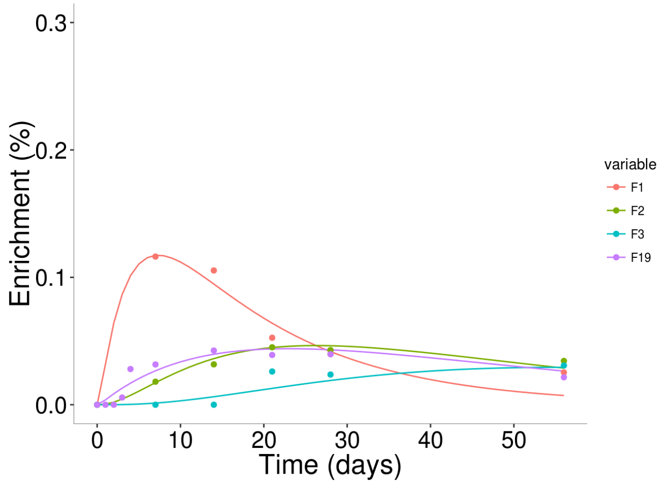	-253.83	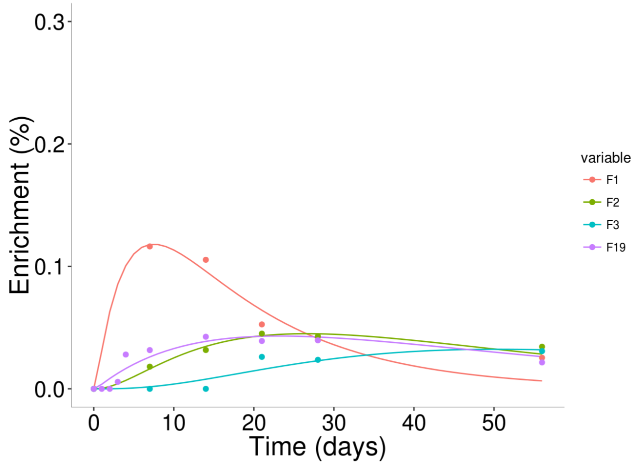

K8	sIgM	-157.3	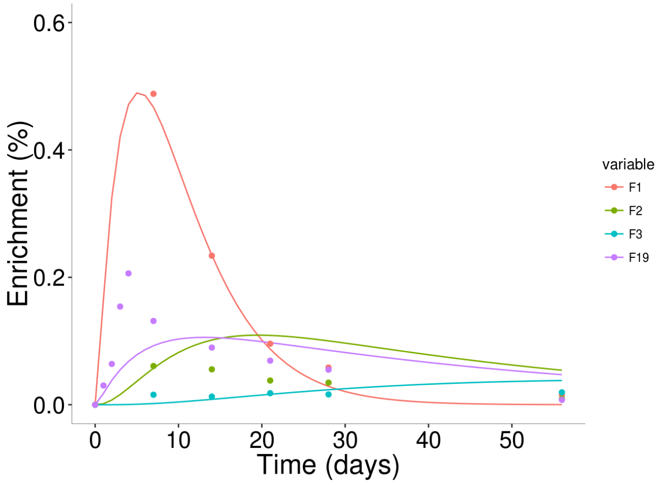	-157.19	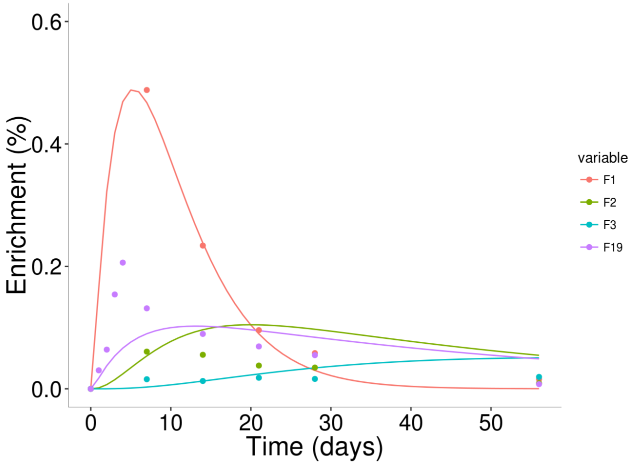
K9	sIgM	-219.77	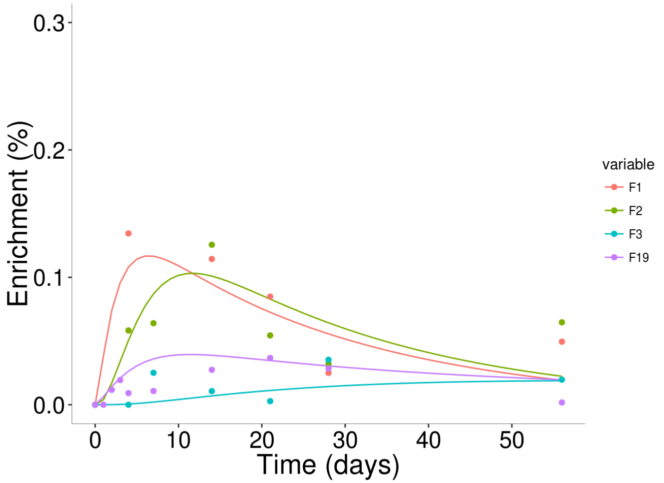	-213.94	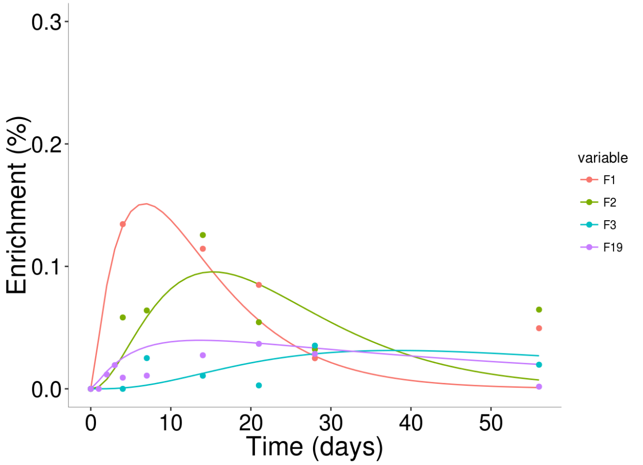

K6	BCR	-214.49	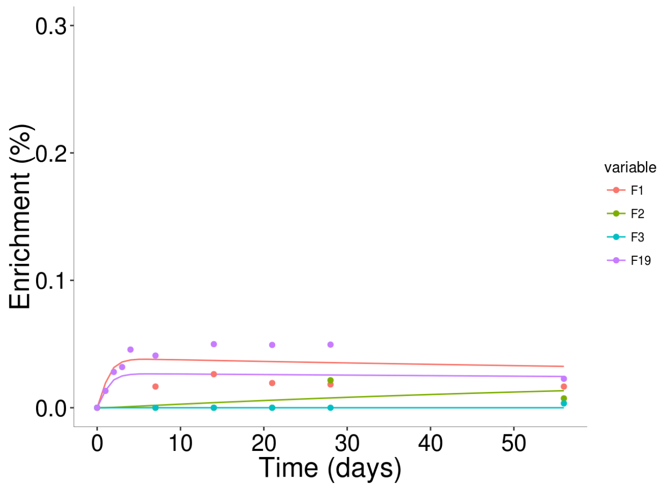	-212.04	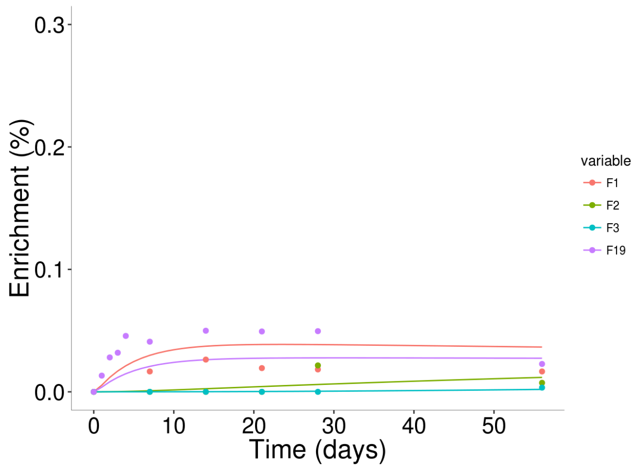
K9	BCR	-235.47	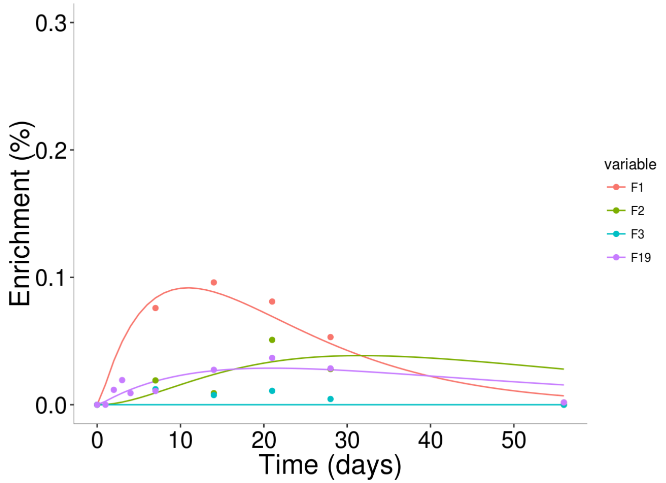	-231.46	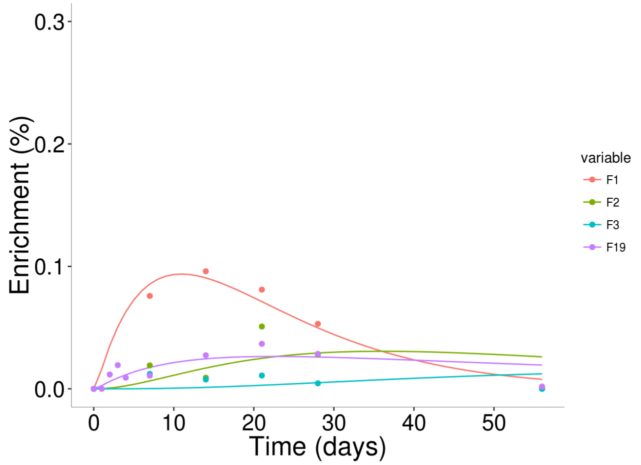

K10	BCR	-216.83	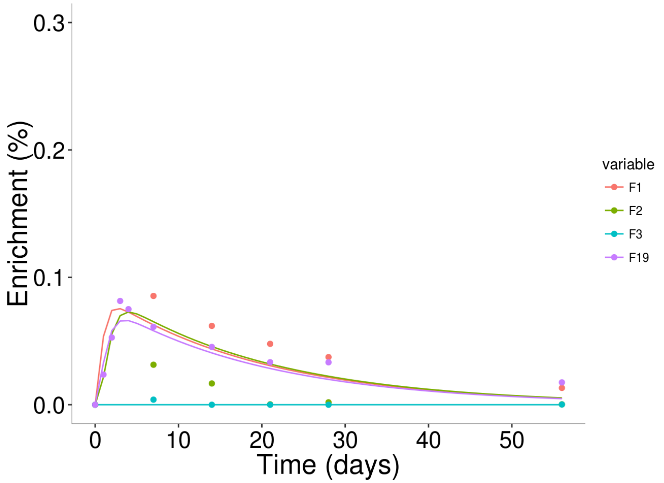	-196.5	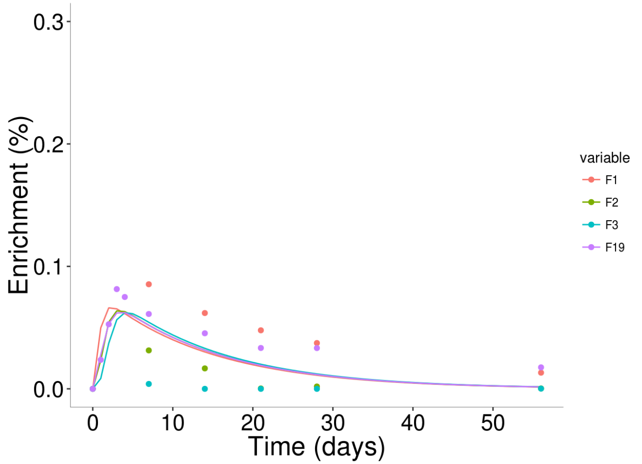
